# Review: Development and Technical Design of Tangible User Interfaces in Wide-Field Areas of Application

**DOI:** 10.3390/s21134258

**Published:** 2021-06-22

**Authors:** Alice Krestanova, Martin Cerny, Martin Augustynek

**Affiliations:** Faculty of Electrical Engineering and Computer Science, VSB-Technical University of Ostrava, 17. Listopadu 15, 708 33 Ostrava-Poruba, Czech Republic; alice.krestanova@vsb.cz (A.K.); martin.augustynek@vsb.cz (M.A.)

**Keywords:** tangible, tangible user interface, smart object, sensors, education, augmented reality

## Abstract

A tangible user interface or TUI connects physical objects and digital interfaces. It is more interactive and interesting for users than a classic graphic user interface. This article presents a descriptive overview of TUI’s real-world applications sorted into ten main application areas—teaching of traditional subjects, medicine and psychology, programming, database development, music and arts, modeling of 3D objects, modeling in architecture, literature and storytelling, adjustable TUI solutions, and commercial TUI smart toys. The paper focuses on TUI’s technical solutions and a description of technical constructions that influences the applicability of TUIs in the real world. Based on the review, the technical concept was divided into two main approaches: the sensory technical concept and technology based on a computer vision algorithm. The sensory technical concept is processed to use wireless technology, sensors, and feedback possibilities in TUI applications. The image processing approach is processed to a marker and markerless approach for object recognition, the use of cameras, and the use of computer vision platforms for TUI applications.

## 1. Introduction

A Tangible User Interface (TUI) is an innovative way of controlling virtual space by the manipulation of physical objects, which are representing digital information. This approach shows a big advantage against a graphical user interface (GUI). A GUI allows the user to work on the computer using a mouse, keyboard, or any special kind of pointing device. However, it usually only deals with one device, and the projection of this device in the virtual space is only a pointer. A TUI is interesting for the user because it directly makes it possible to gain work experience and skills by manipulating a physical object and could give the user a feeling of touch and response [1,2]. A TUI allows the transfer of virtual space into real-world devices. Usually, TUIs work in groups, and the representation of the TUI object in the virtual space is the same or a similar virtual object [3].

This publication presents a descriptive review of TUI methods and their application areas focusing on tangible objects and their technology of recognition. The publication is structured as follows. Section 2 deals with the structure of the review for the description of TUI application areas and technology for the recognition of tangible objects. The period from 2010 to 2020 and a combination of keywords (tangible user interface, TUI, educational smart objects, etc.) were important for the selection of articles. The Scopus, Web of Science, Google Scholar, etc. databases were searched. More information about the review structure is in Section 2—Structure of Review.

The main application groups are teaching, medicine and psychology, programming and controlling robots, database development, music and arts, modeling of 3D objects, modeling in architecture, literature and storytelling, adjustable TUI solutions, and commercial TUI smart toys. In each group, research is described with information about the use of the proposed TUI, the technical solution of the proposed TUI, and the research results. These applications are described in Section 3.

Section 4 contains an analysis of TUI’s technical solutions. The sensory technical solution describes the used wireless technologies for communication and interaction, microcontrollers, sensors, and possibilities for feedback to users, which were used in TUI applications. Image processing describes a marker and markerless approach for object recognition, the used cameras for scanning the manipulation area, and the computer vision algorithms that were used in the TUI applications.

## 2. Structure of Review

This review describes the technical solutions and based concepts of TUI in the wide spectrum of application areas and their description. It focuses not only on the application areas but also shows the technologies used for object recognition. Technology development seems to be the most limiting factor for the further expansion of TUIs into our lives. 

The text contains a review of 64 articles. The articles were selected based on the timespan of 2010–2020. For the review, the articles were selected by different combinations of keywords such as: tangible user interface, TUI, educational smart objects, rehabilitation objects, smart objects, smart cubes, application TUI, tangibles, manipulative objects, and objects recognition. The following databases were used to search for the articles: Web of Science, IEEE Xplore, ScienceDirect, Scopus, Google Scholar.

The unsuitable articles were then excluded based on abstracts and titles. In the final step, duplicity was removed from the selected articles. After the selection, the articles (numbering 66) were selected mainly in the timespan of 2010–2020, however, 11 articles were from the timespan of 1997–2009. The older articles were used for the addition of information on specific terms (TUI, ReacTIVision, etc.) during the processing of the review. The used older articles were innovative and used for additional information to the articles, which build on or were inspired by these older works, e.g., in the section on TUI in architecture. There was a research group that developed this concept gradually.

**Figure 1:**
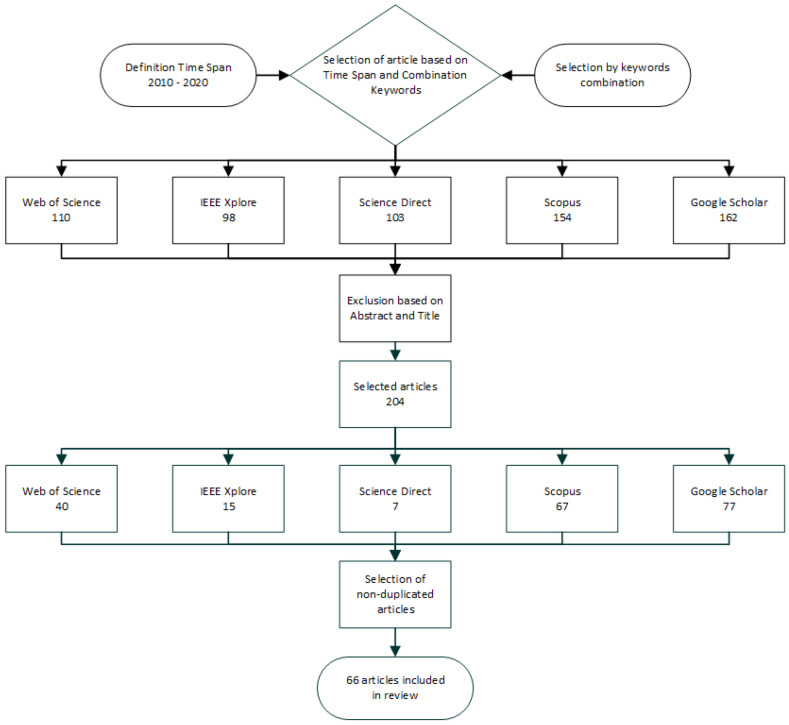
The process of selecting articles for the design and technical solutions of a tangible user interface (TUI).

Figure 1 shows the process of selecting articles for the processing of the review design and TUI technical solution.

In related works, articles can be found describing the past, present, and future of TUI [4]. The difference to previous reviews is that our review mainly represents TUI technologies from the timespan 2010–2020, whereas the previous reviews are from the timespan up to 2009. Other differences are in the definition of the application areas into more specific areas, the different views of descriptions of technical solutions used, such as wireless technologies, sensors, feedback possibilities, image processing, and their marker and markerless approach for object recognition, cameras used for image processing, computer vision algorithms.

## 3. TUI Application Areas

Based on the research, we identified eight basic application areas, which are listed in Table 1. In the following subsections, we focus on each area in more detail. These most common application areas are expanded to two sections focused on adjustable TUI solutions and commercial TUI smart toys applications.

### 3.1. TUI as a Method for Learning

Several findings show that a tangible interface is more advantageous for the learning process compared to classical learning methods. The tangible interface stimulates interest in people, thus they cooperate better. Students explore alternative proposals and become more involved in the class. Problems and the path to a solution are perceived as fun. TUI exposure was more attractive to visitors than classic or multitouch exhibits for acquiring knowledge in institutions such as museums [5,6,7,8,9].

Ma et al. from Exploratorium proposed a new exhibit for the visualization of phytoplankton distribution in the world’s oceans. The technical concept of TUI exposure in the museum looks like this: physical circles with a glass lens are placed on the MultiTaction Cell display, with infrared reflective reference marks in the glass. The MultiTaction Cell detects these markers and displays the specific area closer. It has shown that the exposure is suitable for 8 years old and older. The physical circles were difficult for younger children. Touching a physical circle was a critical precursor to continued engagement with the exhibit. For example, people who interact with this exhibit spent more time there and reacted to the other exhibits where there were virtual circles [10].

Yui et al. from Waseda University proposed a floating TUI to create a perception of animacy (the movement of a polystyrene ball). The following elements are typical for a tangible system: interactivity, irregularity, and automatic movements resistant to gravitational force. Three mechanisms have been implemented: a floating field mechanism, a pointer input/output mechanism, and a hand-over mechanism. The technical concept of a floating field formation consists of a PC, Arduino controller, a polystyrene ball, and a blower fan with a variable flow rate controlled by PWM (pulse width modulation). The floating field is the area where the ball can move. Kinect was used in a pointer input/output mechanism. It can determine the position and shape of the user’s hand and the position of the ball. The hand-over mechanism is used for handing over the polystyrene ball from one fan to another fan. The technical solution is using servomotors. Servomotors can tilt the fans forwards and backward by changing the direction of the ball. The tangible interface was tested in the Museum of Science and once again visitors showed great interest and wanted to interact with it [8].

Vaz et al. from the Federal University of Minas Gerais proposed a TUI in geological exhibitions. The user interface is a display with four power-sensitive resistors and four brightness controls, RGB, LEDs, and sound. Graphical and sound information was implemented among the handling objects. Sound information was used for visually impaired visitors and graphical information for other visitors. These elements were connected to Arduino Leonard, on which the program code ran, and other components included a PC, speaker, and projector. The users were pleased with this exhibition [11]. 

Schneider et al. from Stanford University developed BrainExplorer for the education of neuroscience. BrainExplorer represents each area of the brain with three-dimensional tangible elements. It is used as a new educational tool of neural pathways for students. This learning tool allows students to gain new information through a constructive approach. This approach allows users to associate a technical term with a physical object rather than an abstract concept and to explore several possible connections between these parts and the resulting behavior. The technical solution of this interface is as follows: the polymer replica of the brain can be deconstructed on the board, manipulated, and reassembled. The placed parts of the brain are monitored by a high-resolution webcam. The Reactivision Framework [12] was used to label different parts of the brain. Another camera was placed between the eyes and records what the brain perceives. The projector with a short time shows the connection of the brain from below the table. Finally, users communicate with each connection separately using an infrared pen, whose signal is detected by Wiimote (Nintendo, Redmond, WA, USA). The software is programmed in Java using the existing Reactivision Framework and communication libraries for Wiimote. The foundation of the teaching is relevant, learning theories are important, and this approach to learning is crucial for promoting knowledge building [9].

**Figure 2:**
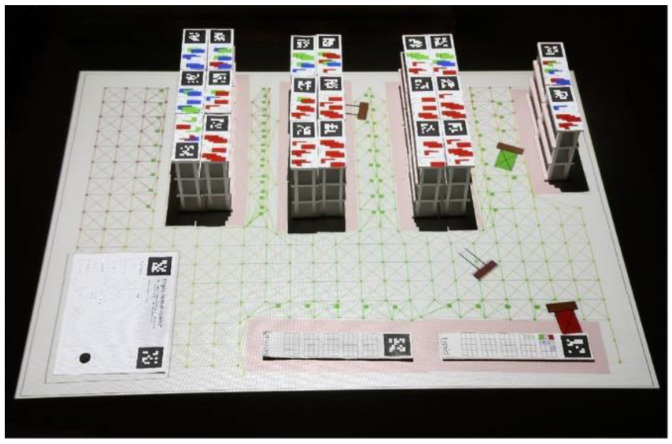
Tinker system for simulation of the warehouse [5].

Schneider et al. from Stanford University developed the TinkerLamp for the education of logistics warehouse design. The TinkerLamp system is used by logistics apprentices and teachers through TUI, which consists of two modalities. The first modality is a small model of a warehouse with miniature plastic shelves, offices, and more. Each element of the small warehouse is marked with a reference mark, which allows automatic recognition by the camera. The projector located above the table works as feedback. If the two shelves are too close to each other, the navigation nodes will turn red. There is not enough space for the forklift. The second interface consists of TinkerSheet, thanks to which it is possible to change the system parameters, start the system, and control the simulation. The user uses a pen or a physical token (e.g., a magnet) to interact. This TUI method allows for a better experience and learning for students than classical designs by pencil and paper. This approach to learning promotes exploration, collaboration, and the playfulness of the task. From the results, the TinkerLamp TUI is better for a problem-solving task than a multitouch interface [5,13,14]. The Tinker system is shown in Figure 2.

Starcic et al. from the University of Ljubljana developed a new method for learning geometry. Geometry was learned through cognitive and physical activity. TUI enables students to support geometry education and integrates them into classroom activities. The concept of the approach combines physical objects and a PC interface. Physical objects with different functionality were placed on the surface. The proposed application displays these objects on the computer screen. When the object is repositioning, the corresponding action is instantly displayed on the screen. From the results of the research, the presumption of learning by the new TUI method was confirmed. TUI using a PC is suitable for students with low fine motor skills or children with learning disabilities when they cannot use rulers, compasses, or a computer mouse [15]. 

Sorathia et al. from the Indian Institute of Technology Guwahati is teaching students with tangible user interaction. Undergraduate and graduate students tested these teaching approaches. The courses were focused on the design and development of an educational system for these topics: mathematics, chemistry, physics, learning fruits, colors, history, astronomy, energy conservation, tourism, etc. Students can choose any topic. Three types of TUI were described in the paper [16]. 

**Figure 3:**
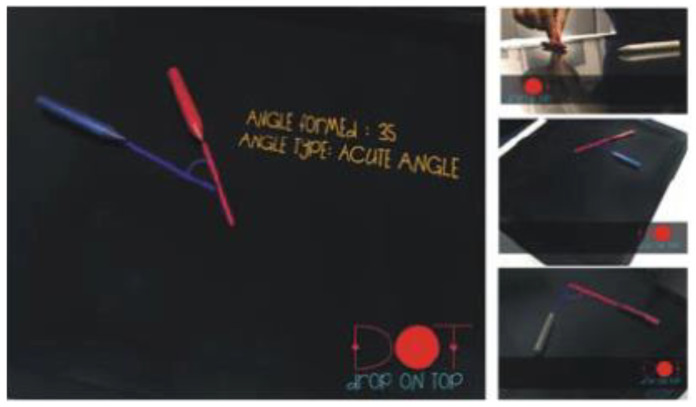
Formation of acute angle [16].

The first type was realized to understand the relationship between the degree of angle and the theory of acute, obtuse, and right angles. It can be used for children aged 8–10 years. The technical concept consists of a tabletop, a camera above the table, a projector under the table. Two different color markers are placed on the table and extended rays are formed from these markers. The angles are created in this way (see Figure 3). The camera detects smart objects (markers), it sends the signal to OpenCV (Open Computer Vision software) [17]. Color lines were shown on the table by the projector after image processing [16]. 

**Figure 4:**
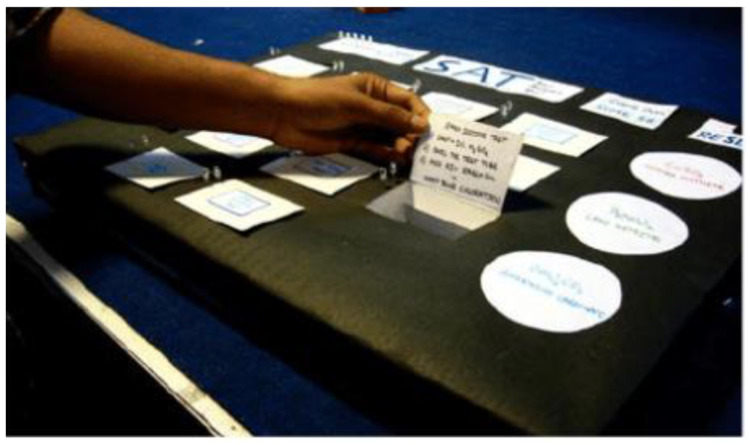
Salt Analysis—student presents reaction details [16].

The second type is for students of chemistry. The students have to determine the cations and anions present in an unknown salt. The technical concept consists of 14 reaction boxes that are placed at the table, the control panel placed on top, and a results section. The results section represents the feedback through LEDs on the control panel. LEDs were controlled by Arduino. Arduino was placed underneath the reaction box (see Figure 4). It was used to identify the saltboxes and correct the student’s reactions [16]. 

**Figure 5:**
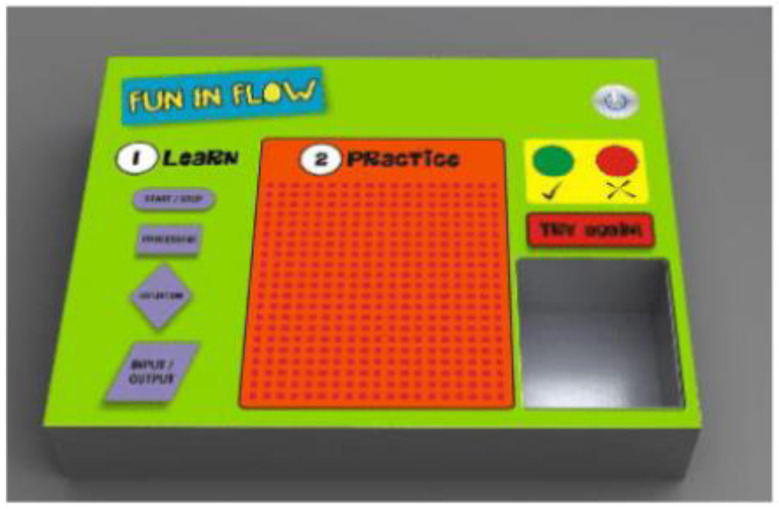
Overall structure of a TUI for learning flow charts and algorithms [16].

The third type is for learning flow charts and algorithms. The technical concepts of the approach are radiofrequency identification (RFID) readers and tags. Students can manipulate predefined symbols to write a program. Symbols are placed in a practice area. A tag is situated in every symbol and the RFID reader is placed below the practice area. Green and red lights indicate positive and negative feedback (see Figure 5). Students showed enthusiasm for the courses where a TUI was used [16]. 

**Figure 6:**
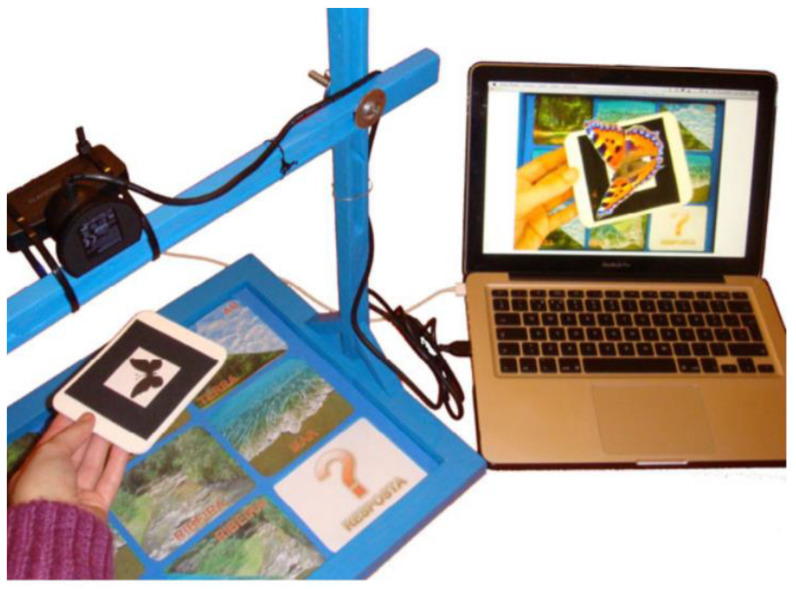
Proposed TUI system with a laptop [18].

Campos et al. from the University of Madeira Interactive Technologies Institute developed a combination of a TUI and augmented reality for the study of animals and their environments (sea, river, land, air). This approach can be used for kindergarten children. The system consists of a wooden board with printed images, which is divided into nine blocks. Each block has its marker. A camera arm is attached to the board, which is connected to a laptop (see Figure 6). This system evaluates the correctness of the image selection. It can be used to arouse greater interest and motivation in new knowledge through games [18]. 

**Figure 7:**
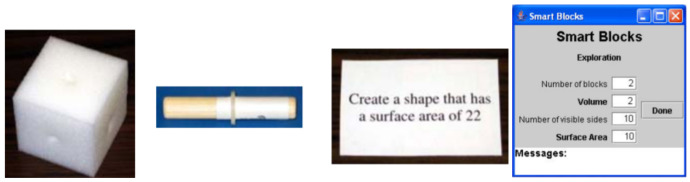
Proposed SmartBlocks, connector, question, a screenshot of the SmartBlocks interface (from the left) [19].

Girourard et al. from Tuffs University proposed Smart Blocks for reinforcing the learning of spatial relationships. Smart blocks are mathematical intelligent manipulative blocks, which allow users to explore spatial relationships (area, volume of 3D objects) and provide continuous feedback to enhance learning. Smart Blocks are composed of 3 lightweight cubes, 3 dowel connectors, a workspace, and cards with questions (see Figure 7). Each cube has 6 holes, where one is situated in the middle of every side. Connectors are situated in holes. Every block, connector, and question contains a unique RFID mark. When the shape is assembled, the user places it on the workspace that contains the RFID reader. The reader recognizes all RFID tags located on the desktop and the basic Java program recognizes identifiers for blocks, connectors, and questions. TUIML (a TUI language) was used for fast modeling to track the structure and behavior of the Smart Cubes.

The TUIML displays the TUI structures of token bindings (as physical objects in digital form) and constraints (physical objects are limited by token behavior). The TUIML has been used to refine modal transitions and define tasks that users can complete while the system is in a particular mode. When the smart blocks are connected, the software can calculate the volume and the surface area of the shape. The information is shown in a GUI. It also displays the number of blocks and visible sides. The proposed system can be an effective teaching tool because it combines physical manipulation and real-time feedback [19].

**Figure 8:**
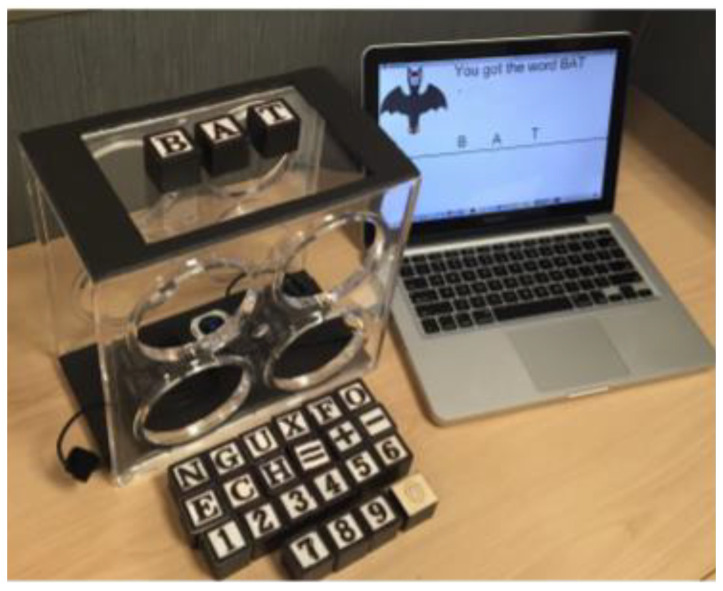
Proposed BlackBlocks with a running example of 3-letter words [20].

Almukadi et al. from the Florida Institute of Technology proposed BlackBlocks to support language and education math. The BlackBlocks were used for the education of children aged 4 to 8 years. The technical concept consists of a laptop with interface software, a transparent box with a web camera, ReacTIVision for recognition of fiducial markers, tangible blocks with fiducial markers on the bottom, and with letters, numbers, or mathematical symbols on the top (see Figure 8). The tangible blocks are placed on the transparent box and the web camera scanned the fiducial markers, ReacTIVision recognized the markers, and the software gave feedback and the next question. The children are motivated in their education and have a positive mood and a good experience. These are the advantages of the proposed tangible system [20].

**Figure 9:**
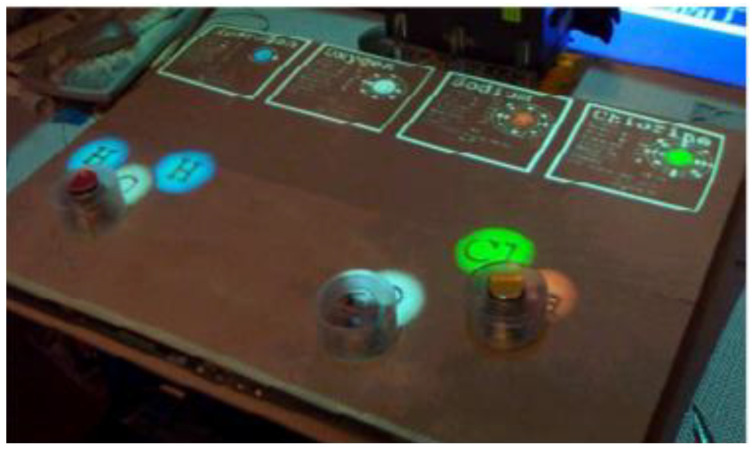
Sensetable for the education of chemical reactions [21].

Patten et al. from MIT Media Laboratory proposed Sensetable for the education of chemical reactions (see Figure 9). Sensetable is an electromagnetic system that tracks the position and orientation of multiple wireless pucks (objects) on a desktop display. The system tracks objects quickly and accurately without susceptibility to occlusion or changes in lighting conditions. Monitored objects can be modified by adding physical numbers and modifiers. The system can detect these changes in real-time. The technical concept consists of commercially available Wacon Intuous tablets. These tablets are placed next to each other and form a scanning area of 52 × 77 cm. These tablets can only scan two objects. The system allows for new interactions between physical objects and digital systems. It can detect the distance between pucks and control the amount of displayed information [21].

Reinschlüssel et al. from the University of Bremen proposed a scalable tangible system for learning algebra. The technical concept consists of a multitouch tablet and interactive tangibles (two different sizes of tiles). Tangibles are placed on the screen. There is positive visual feedback (blue color) when the equation is right and negative visual feedback (red) when the equation is false. Based on testing, the concept of learning algebra is good for students who have some previous knowledge. There is space for improvement for the didactic concept, intuitive color set, and clear strategy [22]. 

Gajadur et al. from Middlesex University, Mauritius, proposed TangiNet. TangiNet is a tangible user interface for teaching the properties of network cables (twisted pairs, coaxial cables, fiber optics). The technical concept consists of a PC under the table with a processing application, a monitor screen on the table, tangible objects, a webcam mounted on a tripod, and a scan place on the table. On the screen, there is an educational text, task, and voice. When the user puts the objects on the screen in the same movement, the digital screen displays the results of the connection of two objects. Tangible objects are miniature laptops, routers, antennas, and TV screens, connectors of coaxial cables, fiber optics, and twisted pairs placed on the plastic circle. These are for learning the properties and connections of network cables. Tangible plastic letters, numbers, and symbols are for the choice of answers during the test. Each tangible has specific markers. ReacTIVision was used for the identification of physical objects. Based on testing, the proposed system is suitable for networking courses [23].

**Figure 10:**
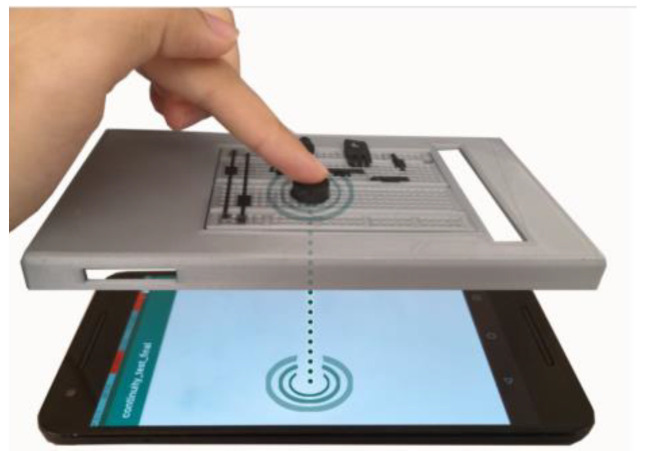
Printed TangibleCircuits with an audio interface on a smartphone [24].

Davis et al. from Boston University proposed TangibleCircuits. It is an education tutorial system for blind and visually impaired users that allows understanding the circuit diagrams. The user has haptic and audio feedback. The proposed system translates a Fritzing diagram from a visual medium to a 3D printed model and voice annotation. Tangible models of circuits were printed using conductive filament (Proto-Pasta CDP12805 Composite Conductive PLA). A 3D printed model of the circuit is affixed to a capacitive touchscreen of a smartphone or tablet for voice feedback. The audio interface of the printed TangibleCircuits is displayed on a smartphone (see Figure 10). When the user touches on the circuits, the application in the smartphone gives audio feedback about the concrete component. The users were satisfied with the system and understood the geometric, spatial, and structural circuits [24]. 

Nathoo et al. from Middlesex University proposed a tangible user interface for the education of the Internet of Things (IoT). The technical concept of the TUI consists of a web camera on a tripod that scans a large multitouch display screen, a light source, and a computer. The screen provides the user with digital feedback. Tangibles are real objects (pulse oximeters, smartphones, sound sensors, motion sensors, etc.) and plastic figures with specific fiducial markers. The computer is the main component with software showing questions, learning text, and processing specific markers from tangibles by ReacTIVision, the TUI allows students to positively learn IoT in a creative and inspirational way [25]. 

### 3.2. Application of TUI in Medicine and Psychology

**Figure 11:**
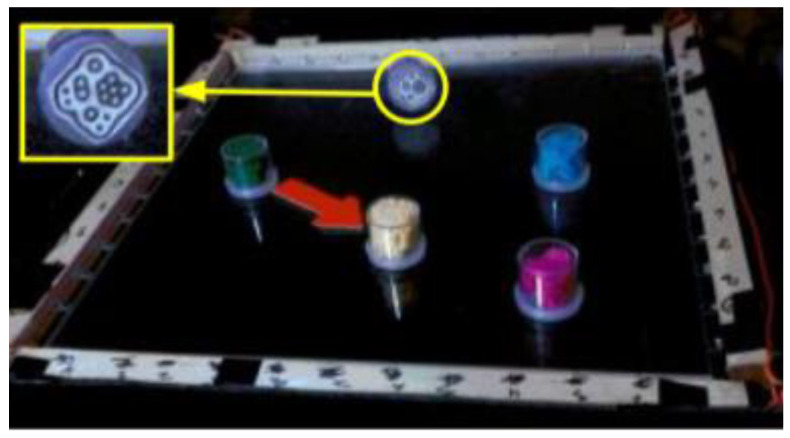
Design of MICOO [26].

Manshad et al. from New Mexico State University proposed a TUI which enables the blind and visually impaired to create, modify, and naturally interact with diagrams and graphs on the multitouch surface. MICOO (Multimodal Interactive Cubes for Object Orientation) objects are a cube system with a multimodal tangible user interface (see Figure 11). This tool allows people to orient and track the position of diagrams and graph components using audio feedback. While the user is working, there are also formal evaluations such as browsing, editing, and creating graphs, and more. The surface is controlled via multiple infrared cameras that monitor the movement of objects using the detection of reference marks. After validation of the mark, it is immediately plotted on a Cartesian plane. The software application was created in C # extending the opensource reacTIVision TUIO Framework [26]. 

**Figure 12:**
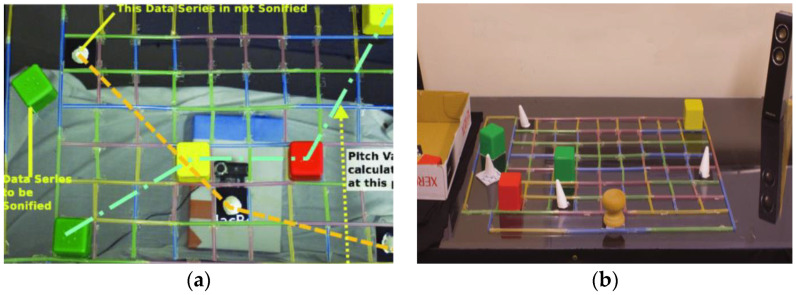
Illustration of the interaction with the Tangible Graph Builder (**a**), Tangible Graph Builder with tangible grid, and tangible objects (**b**) [28].

McGookin et al. from the University of Glasgow proposed a TUI which enables visually impaired users to browse and construct both line and bar graphs nonvisually. The technical concept consists of a tangible grid (9 × 7), two objects with different shapes, and a weight (cube, cone) representing phicons for two data series, and ARToolkit (see Figure 12) [27]. ARToolkit is an augmented reality tool. It can also be used to create graphs for the visually impaired. These tools are used by the camera for rendering 3D space. The marks of individual objects were monitored using a Logitech WebCam Pro 9000. The software for tracking the markers and their determination of position in the grid was written in C#. The results obtained from the testing are comparable to performing similar virtual haptic and auditory tasks by blindfolded sighted users [28].

De La Guía et al. from the University of Castilla-La Mancha proposed a TUI technology for strengthening and stimulating learning in children with ADHD. Children with Attention Deficit Hyperactivity Disorder (ADHD) suffer from behavioral disorders, self-control, and learning difficulties. Teachers and therapists can monitor children’s progress using a software system. The user communicates with the system via cards (or other physical objects) that have built-in RFID tags. This is the principle of this technology. The user selects a card with the appropriate image and zooms in on the mobile device that contains the RFID reader. The loaded electromagnetic signal is processed by the server and sends information back to the device whether the card is selected correctly or incorrectly. Sounds, praise, and motivational messages support the children’s interest in the next game. Games that run on a PC are also displayed on the projector. From the results, it follows that TUI games have a motivational effect and noticeable improvement in children with ADHD. Long-term effects on attention, memory and associative capacity need to be monitored for years [29]. 

**Figure 13:**
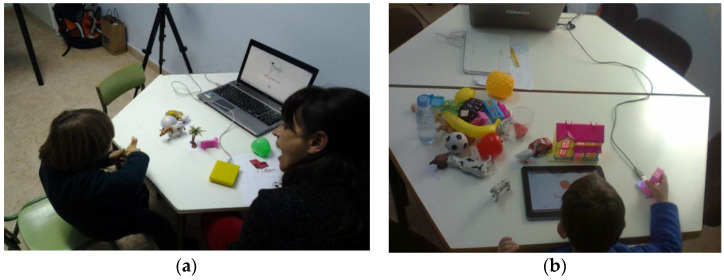
Techear-child session with TUI (**a**), Self-learning using a multitouch interface (**b**) [30].

Jadan-Guerrero et al. from Technological University Indoamerica proposed the TUI method called Kiteracy. Kiteracy enables children with Down Syndrome to develop literary and reading skills. The technical concept consists of plasticized cards grouped into categories (animals, fruit, homes, landscapes), physical letter objects, RFID readers, a computer, and a tablet (see Figure 13). The physical letter can also play sound. The RFID reader is attached to the laptop and it reads the marker from each card and object. The results show that the technology could strengthen speech, language, and communication skills in the literacy process [30].

**Figure 14:**
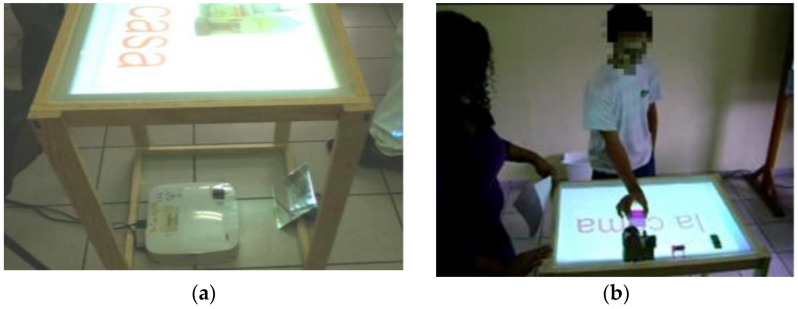
Prototype of tabletop (**a**), User manipulates with tangible object (**b**) [31].

Haro et al. from the University of Colima proposed a book with a tangible interface for improving the reading and writing skills of children with Down Syndrome. The system is composed of a tabletop, projector projected educational materials, webcam scanned tags from tangibles objects, and a multitouch screen in a tabletop, PC with software interface recognized and processed data from a web camera. The user manipulates word and image cards and puts them on the tabletop according to the task (see Figure 14). Each card has unique tags for image recognition. The proposed system is good for analysis by teachers and experts. Children like the system and they were motivated to read and write [31].

**Figure 15:**
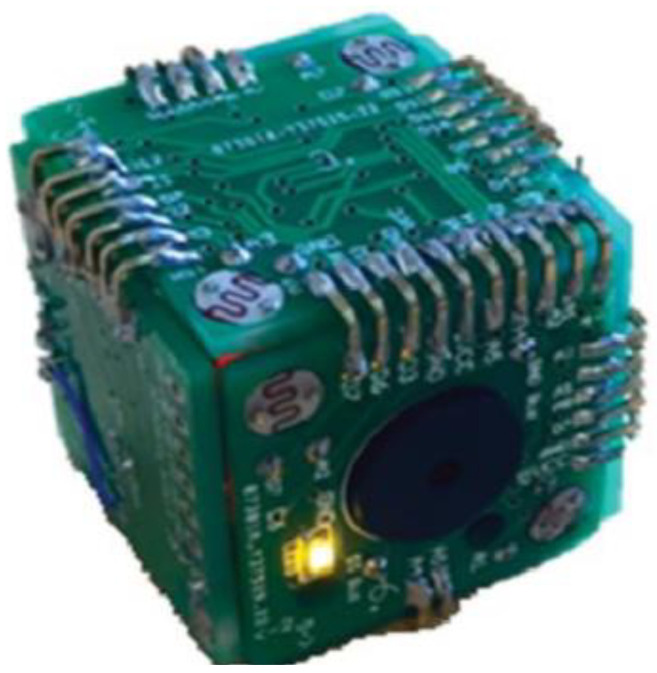
Smart cube for early detection of motoric impairments in childhood [32].

Martin-Ruiz et al. from the Polytechnic University of Madrid proposed a smart toy for early detection of motoric impairments in childhood (see Figure 15). The goal of smart toy research is to improve the accuracy of traditional assessment using sensors placed in common toys, which then provide experts with additional evidence of decision support systems (DSS). Smart Cube includes a buzzer speaker and LEDs to facilitate child stimulation. Each cube has two Light Dependent Resistor (LDR) sensors on its face. An accelerometer with a gyroscope and compass functionality was placed inside each cube. For sleeping and waking the cube, a tilt sensor was included for the detection of movement. The proposed Smart Cubes allow the recording and analysis of data related to the motoric development of a child. Therefore, professionals can identify a child’s developmental level and diagnose problems with the motoric system [32]. 

**Figure 16:**
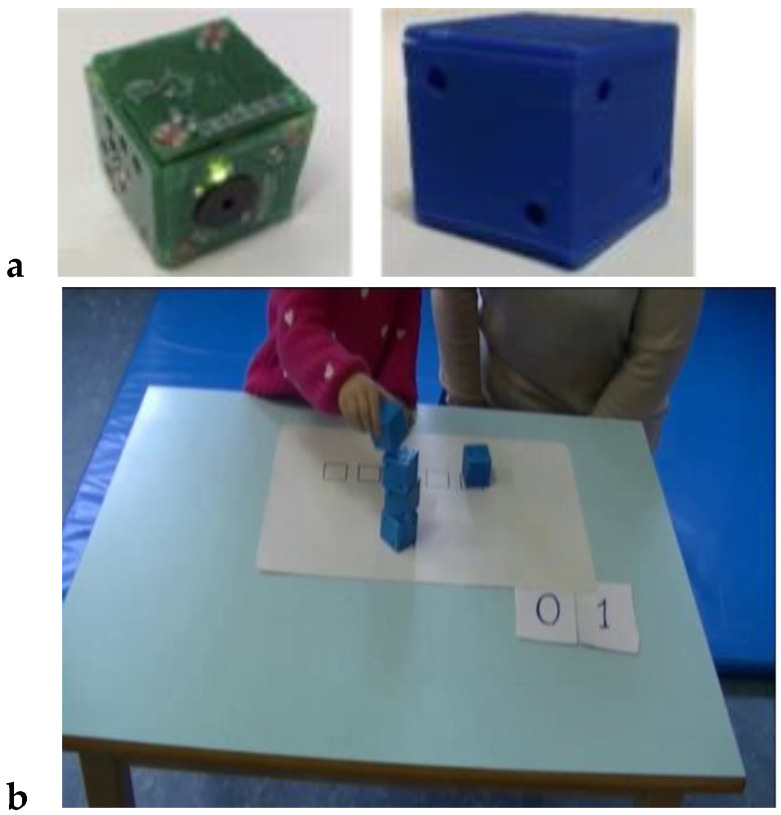
(**a**) Smart toys for detecting developmental delays in children, (**b**) child manipulates with smart cubes to build a tower [33].

Rivera et al. from the University of Alcala proposed smart toys for detecting developmental delays in children. In this research, they focus on the analysis of children’s interactions with appropriately-selected objects. They represent the design of an intelligent toy with built-in sensors. Children perform various tasks and experts are provided with feedback in the form of measured data. One of the first tasks addressed in the article is the construction of a tower (see Figure 16). The technical solution of cubes consists of: a microcontroller, LiPo battery, and associated protection circuits, wireless technology (RF), sensors (accelerometer, Light Dependent Resistor), power switch, and user interface (LEDs, buzzer). The cubes send sensor data to the collector. The collector contains information about the activity such as date, time, the person that is performing the activity, child identifier, etc. After finishing the activity, the collector saves the received data. A tablet was used to control the activity. The obtained data allows the detection of possible developmental motor difficulties or disorders [33]. 

**Figure 17:**
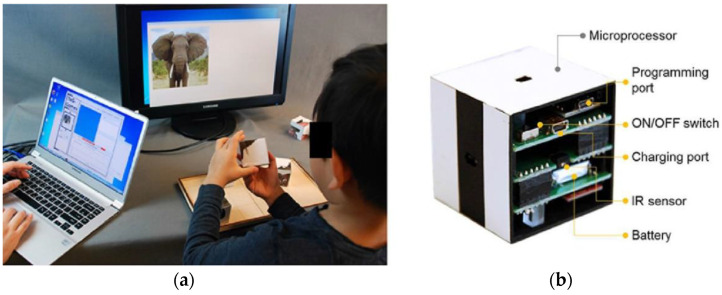
Child using SIG-Blocks with segmented animal faces to match the displayed image and an adult observes the cognitive skills in the graphical user interface (GUI) (**a**), Hardware design of SIG-Block for TAG-Games (**b**) [34].

Lee et al. from Case Western Reserve University proposed TAG-Game technology for cognitive assessment in young children. The children used SIG blocks for playing TAG-Games. SIG blocks are a set of sensor-integrated cubic blocks, and a graphical user interface for real-time test management (see Figure 17). SIG blocks are systems with embedded six optical sensors, a three-axial accelerometer, ZigBee module, and timer in the microprocessor in each SIG block. The system allows the recording of the total number of manipulation steps, correctness, and the time for each. Based on the results from testing TAG-Games, it shows potential use for assessing children’s cognitive skills autonomously [34]. 

Al Mahmud et al. from Swinburne University of Technology proposed POMA. POMA (Picture to Object Mapping Activities) is a TUI system for supporting the social and cognitive skills of children (3–10 years old) with autism spectrum disorders in Sri Lanka. The technical concept of the TUI system consists of tangibles, objects (animals, vegetables, fruit, shapes), and a tablet with the software application. Each plastic toy was pasted with conductive foam sewn with conductive threads on Acryl Felt sheets. Each toy has a specific pattern of conductive foam on the bottom layer of the toy; therefore, each toy has a specific touch pattern. The software application runs on an iPad. There were four activities (shapes, fruit, vegetables, animals) and six levels. The user sees the task in an application and puts the object on the iPad. The software evaluates the correctness of the choice based on multi-touchpoint pattern recognition. The children can play together on a split-screen or alone on a full screen. This system shows that children with mild ASD can play alone to the second level, but children with moderate ASD need more help and time in the initial levels [35].

**Figure 18:**
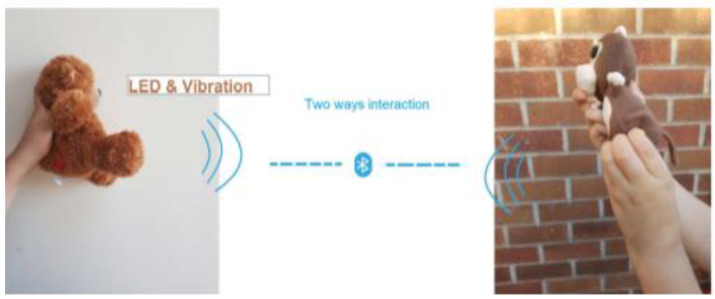
Two children playing with TangToys [36].

Woodward et al. from Nottingham Trent University proposed tangible toys called TangToys for children’s communication about their wellbeing through toys. Each toy has a few embedded sensors, a microcontroller, and a microSD card for recording all interactions. The toys can communicate with one another through Bluetooth 4.2 with a range of 50 m in real-time. The soft ball motion sensor includes a 9-Degree Of Freedom Inertial Measurement Unit (9-DOF IMU), touch sensor (capacitive sensors), and multicolored LEDs for visual feedback. The cube is a printed 3D cube with a motion sensor (9-DOF IMU), touch sensor (capacitive sensor), heartbeat sensor, electrodermal activity sensor, and haptic feedback. Two soft teddies include 9-DOF IMU capacitive touch sensors. The first teddy has visual feedback and the second teddy has visual and haptic feedback. Torus is a printed 3D Torus with an embedded heart rate sensor, electrodermal activity sensor, 9-DOF IMU, capacitive touch sensor, and haptic feedback. When children play with the toys, the sensors sense touch, motion, heart rate, or electrodermal activity and the toys based on these parameters give visual feedback (happy, sad). Toys generate haptic feedback, giving feelings about someone’s presence when a child is not happy. The children can share their well-being with the toys together because the toys can communicate through Bluetooth, and haptic and visual feedback can be actuated on a friend’s device (see Figure 18). In the future, toys can be used in schools for young children or parental monitoring by a mobile app [36].

**Figure 19:**
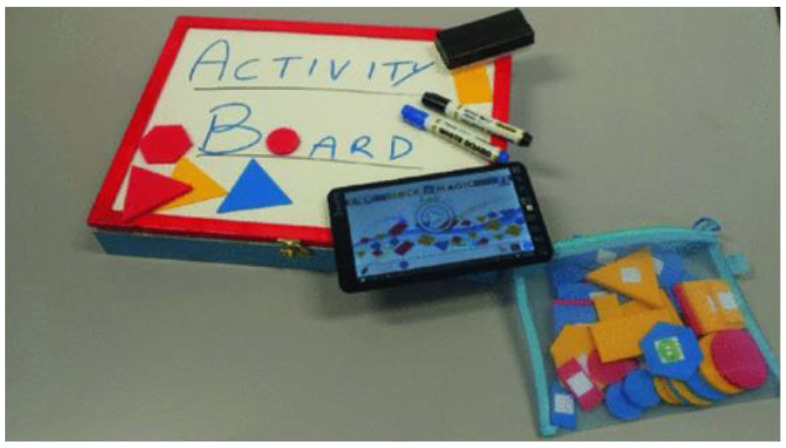
Activity Board 1.0 consists of a wooden box with a radiofrequency identification (RFID) reader and a RFID antenna, tablet, and tangible objects with RFID tags [37].

Di Fuccio et al. from the University of Naples Federico II proposed Activity Board 1.0 for educational and rehabilitation purposes of children 3–7 years old. Tangible objects are tagged with a specific RFID tag and they are detected and identified by an Activity Board (see Figure 19). The Activity Board is a box with a writable whiteboard, inside which is situated an RFID reader (BlueRFID HF), RFID antenna (Antenna HF 310 mm × 180 mm), the main controller (RX/TX module USB/W-Fi), Li-Po battery 3000 mAh with battery module and USB port for charging. The main device can be a tablet, PC, or smartphone. They can be connected to the Activity Board by Wi-fi or USB. The software in the main device controls the Activity Board. The tangible objects are letters, numbers, blocks, dolls, jars with perfume, jars containing candies with a specific flavor. Each object has a specific RFID tag and the RFID reader reads the tag and the software evaluates the process. The software offers different activities. The concept allows connecting different active boards to the main devices [37].

### 3.3. TUI for Programming and Controlling a Robot

The TUI environment is also suitable for understanding the basics of programming. This attitude is described in this subsection. 

**Figure 20:**
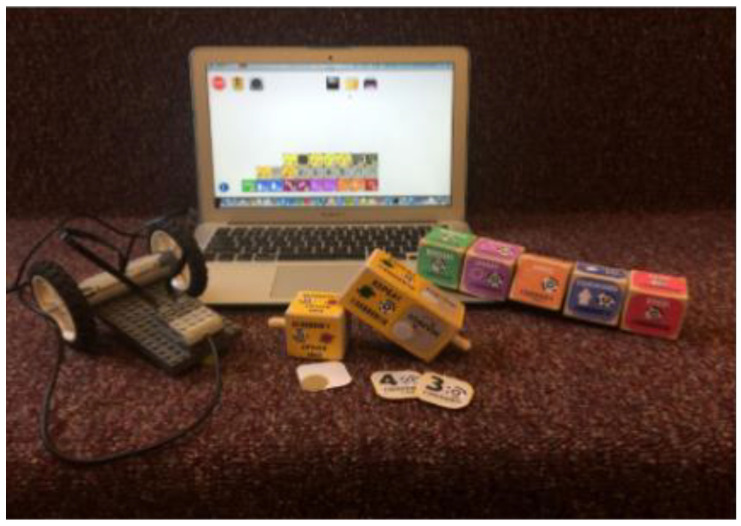
Creative Hybrid Environment for Robotic Programming (CHERP) tangible blocks, LEGO WeDo robotic kit with computer screen shows CHERP graphical user interface [38].

Strawhacker et al. from Tufts University examined the difference in the tangible, graphical, and hybrid user interfaces for programming by kindergarten children. Children used a tangible programming language called CHERP (Creative Hybrid Environment for Robotic Programming) for programming a Lego WeDo robotics construction kit (see Figure 20). It consists of tangible wooden blocks with a bar code. Each block has a command for the robot such as spin, forward, shake, begin, end, etc. The robot has a scanner, it reads the codes and sends the program to the robot. This system was used for kindergarten children for programming. Tangible programming languages can relate to an enhanced comprehension of abstract concepts (for example loops). However, more investigation needs to be done on the learning pattern of kindergarten children [38].

**Figure 21:**
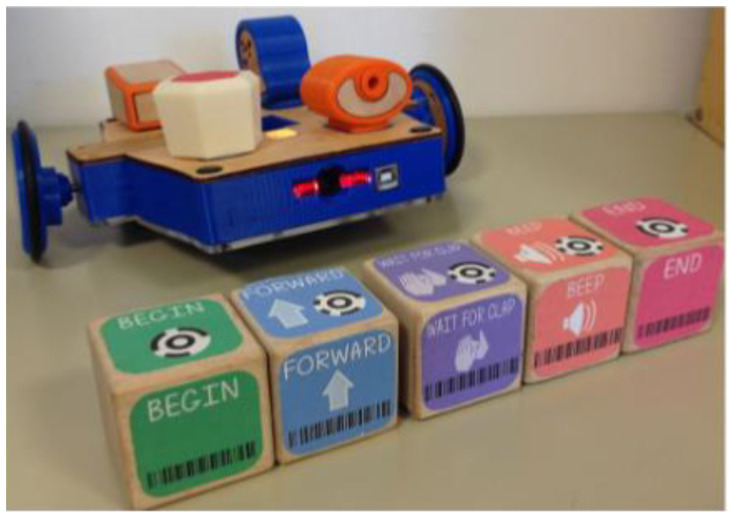
Kiwi robotics kit and CHERP programming blocks [39].

Sullivan et al. from Tufts University worked with the KIWI robotics kit combined with the tangible programming language CHERP, the same as in previous research (see Figure 21). The programming skills were monitored in the pre-kindergarten class for 8 weeks. From the results, it follows that the youngest children can program their robot correctly [39].

**Figure 22:**
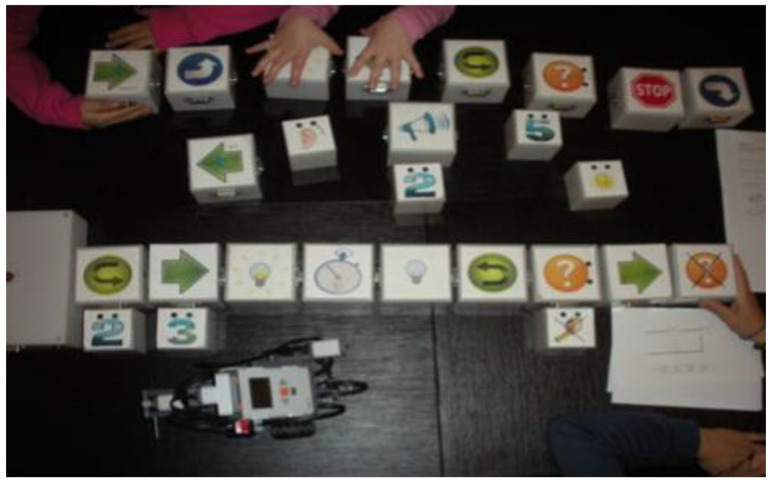
Tangible programming blocks for programming LEGO NXT robot [40].

Sapounidis et al. from Aristotle University developed a TUI programming system for a robot by children. The system consists of 46 cubes, which represent simple program structures and can be connected in the form of programmed code. This code programs the behavior of the Lego NXT robot (see Figure 22). The program is started when the user connects to the master box of the command cube. After pressing the Run button, the master box is started and communication between the connected cubes is initiated. Communication between the programming blocks is based on the RS232 protocol. Each cube communicates with two adjacent cubes. Data was sent to the first cube and received data from the last line. The master cube is used to read the programming structure and to send the program to the remote PC by Bluetooth or RS232. The PC records database information about commands and parameters. The PC completes the recording and compilation and sends it to the Lego NXT robot using a Bluetooth program. The results show that tangible programming is more attractive for girls and younger children. On the other hand, for older children with computer experience, the graphical system was easier for programming [40]. 

**Figure 23:**
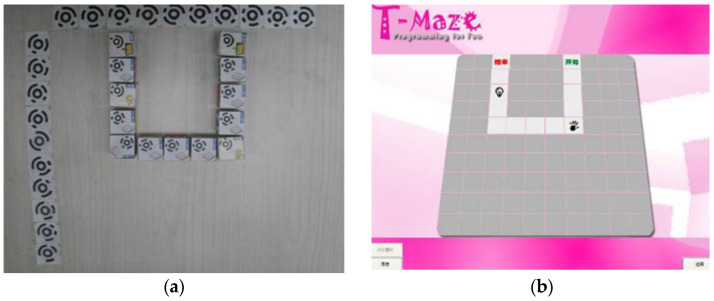
Creation of Maze Area by blocks (**a**), the user interface of Maze Creation (**b**) [41].

Wang et al. from the Chinese Academy of Sciences developed a tangible programming tool called T-Maze. T-Maze is a system for the development of the cognitive and knowledge skills of children aged 5 to 9 years. Thanks to this system, children can understand the basics of programming. The advantage, as with most such systems, is that this process is associated with entertainment. It increases the children’s interest and comprehension of the problem. The whole concept consists of programming wooden blocks with the command (Start, End, direction blocks, loop blocks, sensor blocks), a camera, and a sensor inside the device. The maze game contains two parts: a tool for creating a maze and an escape from the maze. The child creates the program using wooden cubes, which are scanned by a camera, and the semantics of the program are analyzed (see Figure 23). The sensors of temperature, light, shaking, and rotation inside the device are built on the Arduino platform. This solution improves the children’s logical thinking abilities and increases the efficiency of learning [41]. 

**Figure 24:**
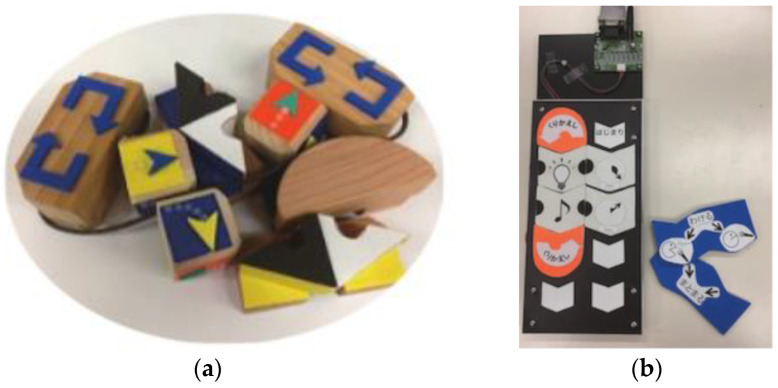
Programming P-CUBEs (**a**), Pro-Tan: programming panel and cards (**b**) [41].

Motoyoshi et al. from Toyama Prefectural University proposed a TUI called P-CUBE. P-CUBE is used as a tool for teaching basic programming concepts to control a mobile robot (see Figure 24). The design concept consists of a mobile robot, a programming panel, programming blocks: a motion block with RFID tags on each face representing movement and instructing the robot to move (forward, back, rotate), timer block (set the movement duration), IF block (two infrared sensors mounted in the mobile robot) can create a line trace program, LOOP block corresponds to WHILE function (repeats the movements of blocks positioned between two LOOP blocks). Different positions of cards and cubes with RFID tags are used for programming. This is all without a PC. The user places wooden blocks on the programming panel. The system uses LEDs, batteries, an infrared receiver, and transceiver, and wireless modules. The system is composed of three block types: initial and terminal blocks, sensor blocks, and motion blocks. RFID (radiofrequency identification) is used for detecting cards and cubes. Using this technology is suitable, but it is not exactly like a microcontroller. Users can create a program simply by placing programming blocks equipped with RFID tags on the program board [42].

Motoyoshi et al. from Toyama Prefectural University developed Pro-Tan to improve TUI accessibility, as well. It consists of a programming panel and programming cards as in the previous research (see Figure 24). This system contains an extra PC. The user creates programs and controls the blinking of the LED light and buzzer sound via Arduino Ethernet. The PC transfers information to a controlled object (robot) by Wi-Fi. The result is that the proposed systems (P-CUBES, Pro-Tan) have the potential for utilization without the supervision of an instructor [42].

Kakehashi et al. from Toyama Prefectural University proposed the improvement of P-CUBE for visually impaired persons. This improvement was the work of the same team as the previous research (see [42]). In this version, there is a programming matrix and wooden blocks with RFID tags. The wooden blocks are inserted into the arrays of the matrix. The programming wooden blocks have tangible commands. For example, plastic-specific arrows placed on the top of wooden blocks represent directions like forward, right, left, backward. After programming the PC transfers, the programming codes to the robot. The disadvantage of this system is the similarity of BEGIN (IF, LOOP) blocks and END (IF, LOOP) blocks. These blocks are represented by oppositely oriented arrows, so it is difficult to distinguish from each other for visually impaired persons. It was necessary for the revision of these blocks [43]. 

**Figure 25:**
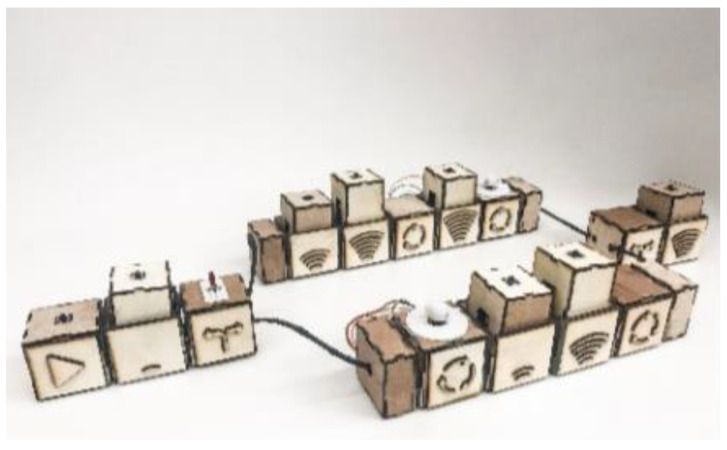
CodeRythm blocks [44].

Rong et al. from City University Hong Kong proposed tangible cubes for the education of basic programming concepts by creating melodies. The toolkit was called CodeRythm and was proposed for young blind and visually impaired students. CodeRythm consists of wooden blocks with magnets for attaching (see Figure 25). The blocks have a specific tactile symbol for the better orientation of students. The blocks can be divided into syllable blocks (do, re, mi, fa, so, la, ti) and distinctive function blocks (Start, Switch, Loop, Pause). Each block includes an Arduino Mini Board, a speaker for audio feedback immediately [44].

Nathoo et al. from the School of Science and Technology proposed a tangible user interface for teaching Java fundamentals to undergraduate students. Students can teach variables, conditions, and loops in the proposed game. The technical concept consists of a graphical user interface with game quests and possibilities and tangible objects to select an answer/option. Tangible objects were plastic letters, numbers, and symbols on cardboard, an arrow was made from cardboard, and real objects such as a small bucket, knob, and a wooden plank. Each tangible object has its own specific unique fiducial mark. A web camera was mounted on a tripod and scanned the table. ReactiVision and Processing processed the scanned fiducial marks. The system is acceptable for teaching programming and there is an option for improvement [45].

**Figure 26:**
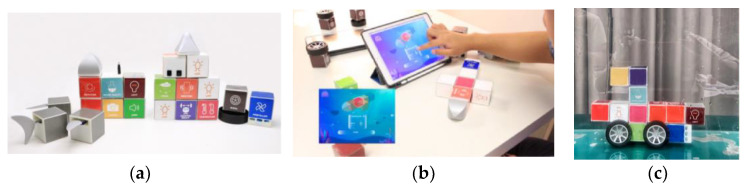
Sensor, actuator and shape modules (**a**), programming and controlling modules (**b**), construction of underwater vehicle (**c**) [44].

Liu et al. from Zhejiang University proposed a tangible and modular toolkit for creating underwater robots called ModBot. Children aged 5–10 years can create the robot and control it for exploring the water environment. It is a good way to understand the knowledge about underwater robots. The ModBot toolkit consists of hardware and software (see Figure 26). ModBot includes 30 hardware modular cubes with different functions: 6 sensor modules (camera, water quality detection, infrared, sonar, temperature, and light sensor), 15 actuator modules (4× LED, 2× rotator, horn, vibration, 3× propeller, 4× wheel), 5 shape modules of the robot (2× head, tail, 2× fin) and 4 counterweight modules for changing the weight of the robot. Each module is waterproof, plastic with an embedded magnet for assembling modules effortlessly. The electronic modules include a lithium battery and one side of the cube is used for wireless charging with a charging platform and an nRF24L01 module for connecting one sensor with 6 actuators. The electronic cubes include a Bluetooth communication board for transmitting data between the tablet and modules. The software application was designed for tablets. The application makes it possible to learn the concepts of modules, assess the balance of the robot based on the weight of modules, show animations, construct with a balance adjustment, and program the modules with the color of the LED. The application shows the results from sensors such as water quality and temperature. The proposed system allows learning, experiencing interesting comprehension in practice, and motivates children to explore the ocean environment. In the future, it will use acoustic communication and test in a real water environment [46].

**Figure 27:**
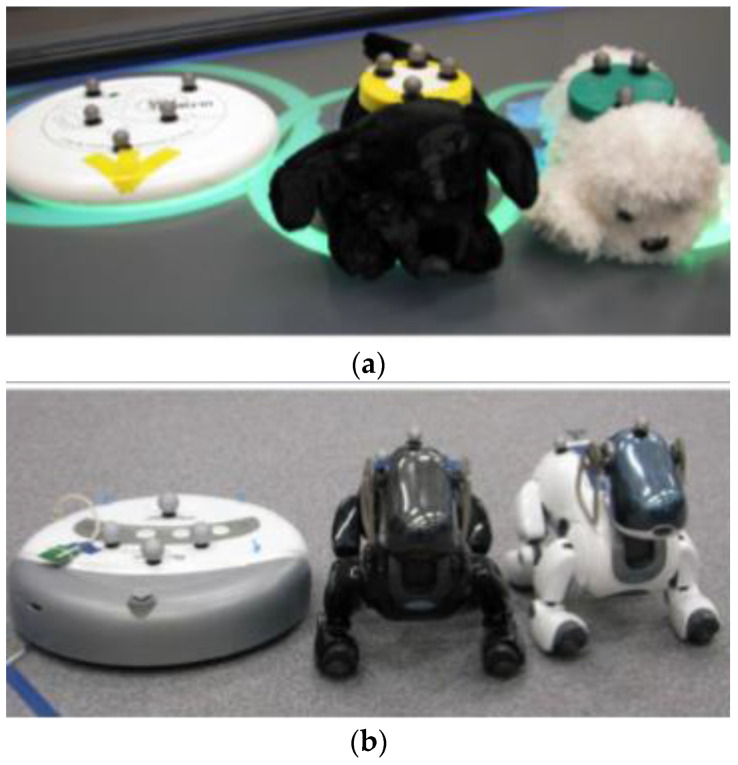
Tangible user interface (**a**), their corresponding robots (**b**) [47].

Merrad et al. from the University Polytechnique Hauts-de-France proposed a tangible system for remotely controlling robots in a simulated crisis (contaminated zone, fire zone, battlefield). Robots can be used in situations when it is impossible to access or dangerous to send a human to the crisis zones. The system was tested in two rooms. In the first room, there were situated tangible objects (small toys or mini-robots representing real robots) with RFID tags, a tabletop equipped with a layer of RFID antennas, data was transmitted by cable to PC 1 (see Figure 27). In the second room, there were situated real robots on the ground, XBee was used for wireless communication to send/receive information about the positions of real robots to the PC 2, a smartphone camera on each robot streams the robot’s surroundings (see Figure 27). PC 1 and PC 2 send/receive data by WLAN/Wi-Fi. Users can control the real robot in real-time by displacing mini robots on the tabletop surface. The proposed system allows effectively remotely controlling of more than one robot. It is better than touch controlling in terms of rapidity, usability, effectiveness, and efficacy. Remotely controlling two robots by tangibles compared to a remote touch user interface is more effective, however, for remotely controlling one robot, the efficiency dimension is unchanged, but controlling the robot was more satisfying for the user than with touch control [47].

### 3.4. TUI for Construction of Database Queries

**Figure 28:**
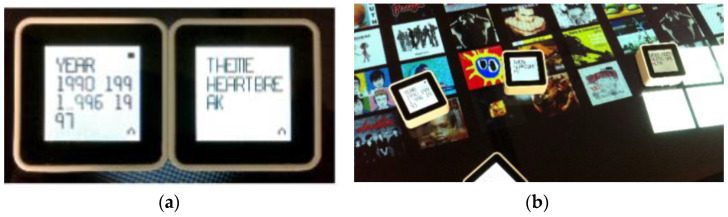
Creation of database Query with Sifteo Cubes (**a**), representation of the results of a query (**b**) [49].

Langner et al. from Technical University Dresden used CubeQuery as a physical way to create and manipulate a musical dataset using basic database queries. CubeQuery uses Sifteo cubes (respective Siftables are commercially available tangible user interfaces developed by MIT Media Laboratory) [48] and a commercially available interactive game system SDK to create applications. It consists of displays, a large number of sensors, and wireless communications. This interactive installation is designed for individual browsing. It allows users to explore the contents of the database (see Figure 28). Each parameter of the search request is tangible. CubeQuery allows the creation of simple Boolean query constructions [49].

**Figure 29:**
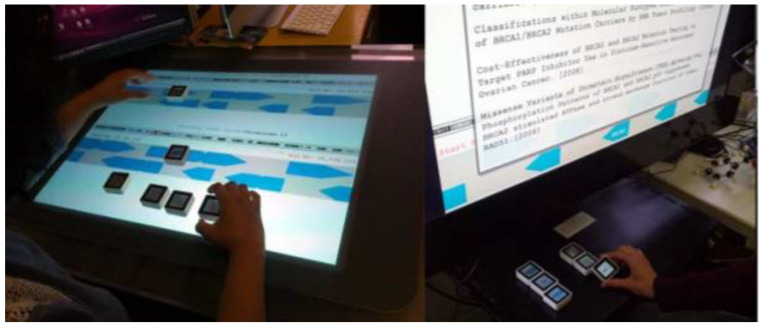
Using Sifteo Cubes for construction of complex database queries [50].

Valdes et al. from Wellesley College used Sifteo Cubes as an active token for the construction of complex queries for large data. Commands for working with data were defined by gestural interaction with Sifteo cubes (see Figure 29). Gestures were specified with the surface, in the air, on the bezel. Active tokens were combined with a horizontal or vertical multitouch surface. This research has a disadvantage because there is an absence of guiding cues feedback [50]. 

**Figure 30:**
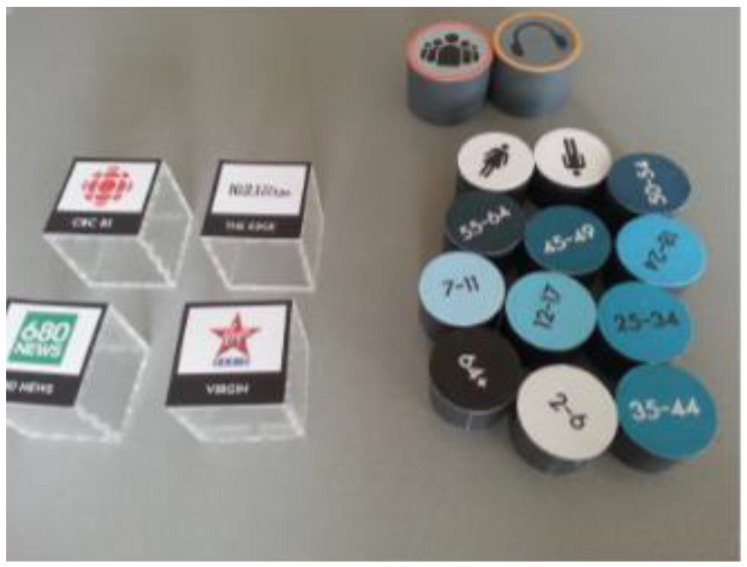
Tangible objects for creating data queries [51].

Jofre et al. from OCAD University proposed a TUI for interactive database querying (see Figure 30). Radio station listenership was used as a testing database. It is computer vision software. The technical concept consists of tokens (radio stations, number of listeners, gender, age of listeners, minutes listened), tabletop, screen display, ReacTIVision software, and visualization code. The users placed queried objects on or off the tabletop surface. The tokens have markers, a camera below the table scanned the markers, and ReactTIVision [12] identified the tokens. The screen display is situated on one end of the table and it displays the output of data visualization. This approach encourages data exploration as a group/team activity. It increases user engagement with the solution [51]. 

### 3.5. TUI in Music and Arts

The development of social skills by music is another area where TUI is used. Villafuerte et al. from Pompeu Fabra University developed a tangible musical interface that enables children with autism to increase social interactions during sessions with other children, even with a nonverbal basis. The TUI system represents a circular tabletop, called Reactable (Reactable Systems S.L, Barcelona, Spain). Reactable is commercially available. Users communicate by direct contact with the table or through objects called pucks grouped into four categories: generators, sound effects (audio filters), controllers, and global objects. Several sessions were held with the children, first following the instructions of the therapist and then playing freely. Everything was scanned by the camera to further analyze the target behavior related to social interaction. This tool can support teamwork in children [52]. Xambó et al. used Reactable, too. They used a tangible music interface for social interaction in public museums [53].

In another approach, Waranusast et al. from Naresuan University proposed a tangible user interface for music learning called muSurface. The main idea of hardware is based on ReacTable, but its computer vision algorithm was implemented. Children can use tangible musical symbols to compose a melody, which is then played through the computer’s speakers and the corresponding effects are placed on the surface. The technical solution consists of music tokens (whole/half/quarter notes, etc.), an infrared camera, projector, speakers, mirror, table, display surface (rear diffusion illumination system), and infrared light sources attached to the corners of the table. The main principle of the solution is rear diffusion illumination, which works when tokens touch the table surface. The infrared light from the illuminators is reflected and captured by an infrared camera. The camera is situated in the center of the table. The projector was used as visual feedback. The infrared image was transformed into user interface events by computer vision algorithms. The mirror allows camera projector calibration because it creates a distorted projected image onto the surface. Therefore, in this way, the projector’s coordinates differ from the camera’s coordinates and compute the homography of these points. The binary image was made from the infrared image by image preprocessing. It was used for the classification of the image based on the K-means method of the nearest neighbor (KNN) using 9 features. These features were extracted from each connected object. Afterward, each region is classified to each note based on KNN. The distance transformation is used to find the localization of each musical object on the five-line staff. The students give positive feedback to the system. They can touch, manipulate, and play with musical notes simply over the didactic method [54]. 

**Figure 31:**
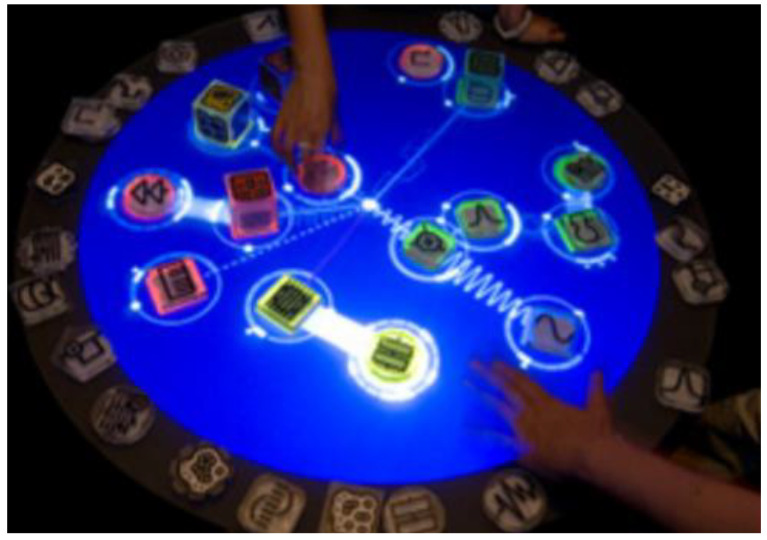
Spyractable consists of: Reactable and tokens with tags in action [55].

Potidis et al. from the University of the Aegean proposed a prototype of a modular synthesizer called Spyractable. The technology is based on a table musical instrument called “Reactable” and computer vision ReacTIVision (see Figure 31). An integral part of this instrument is a translucent round table. The hardware consists of a Plexiglas surface, 45 infrared led lights, a camera without an infrared filter, an infrared light pass filter 850nm, a projector, and a laptop. Users communicate by moving tokens (two amplifiers with envelope control, one time-delay effect, one chorus/phase, one mixer, three oscillators—violin, trombone, trumpet, etc.), changing their position, and invoking an action that changes the parameters of the sound synthesizer. A projector under the table renders the animations. Each token has its special tag, which is read by a camera located under the table surface. The computer vision ReacTIVision reads the token ID and the software evaluates the information about each symbol, position, time, etc., and then plays the compiled music. This technology was interesting for users. However, it needs more testing with complicated modules [55]. 

**Figure 32:**
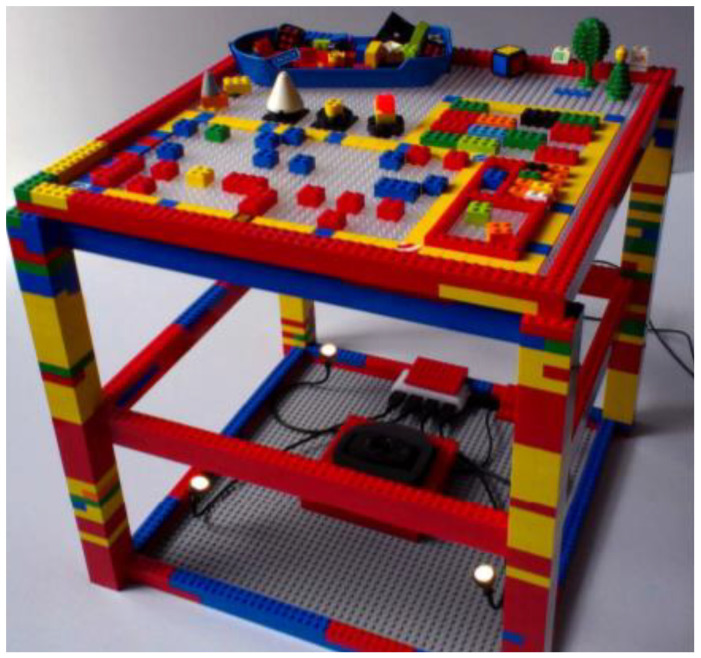
A tangible rhythm sequencer with parameter controls, camera, illumination [55].

Gohlke et al. from Bauhaus University Weimar proposed a TUI for music production by Lego Bricks. Interface widgets (Lego tokens) were created from Lego bricks, tiles, and plates. They are constructed as rotary controls, linear sliders, x/y pads, switches, and grid areas. The technical concept consists of Lego bricks, a backlight translucent Lego baseplate, camera, OpenCV library. Lego tokens are put on the Lego baseplate and scanned by the camera (see Figure 32). An OpenCV library is used for image processing in real-time. The position, color, orientation, and shape of the bricks are tracked using the camera. The system allows the creation of rhythmic drum patterns by arranging colored Lego tokens on the baseplate [56].

### 3.6. TUI for Modeling 3D Objects

**Figure 33:**
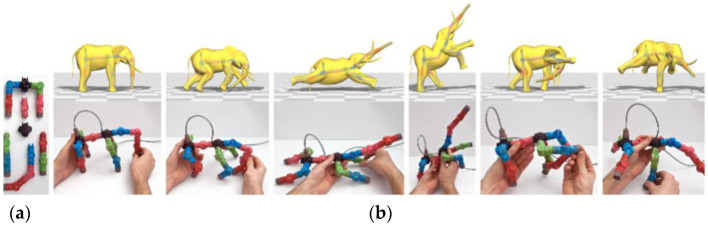
Modular, interchangeable parts for construction skeleton of elephant (**a**), manipulates with elephant (**b**) [57].

Jacobson et al. from ETH Zurich proposed a tangible interface to building movable 3D objects (see Figure 33). The technical solution consists of mechanically moveable joints with built-in sensors to measure three intrinsic Euler angles. It is for editing and animation in real-time. During assembly, the device recognizes topological changes as individual parts or before the assembled subtrees. Such semi-automatic registration allows the user to quickly map the virtual skeletal topology of various characters (e.g., alligators, centaurs, ostriches, and more). The user assembles the skeleton from modular parts or nodes in which there are Hall sensors for measuring Euler angles. For larger nodes, rotary movements are monitored using LEDs and photosensors. Each connection contains a dedicated microcontroller. The whole instance can be understood as a reconfigurable network of sensors. Each connection acquires angular data locally and communicates via a shared bus with the controller. Compared to a classic mouse and keyboard, the TUI shows better results. The device also provides input for character equipment, automatic weight calculation, and more [57].

**Figure 34:**
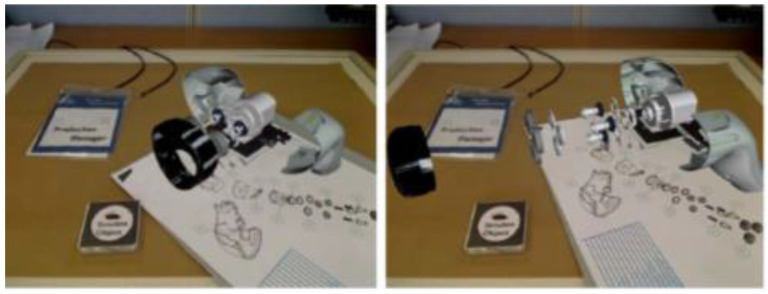
RFID-based tangible query for role-based visualization [58].

Lee et al. from Chonnam National University proposed a tangible user interface for authoring 3D virtual and immersive scenes. Augmented reality with a TUI interface enables interaction with virtual objects naturally and intuitively and supports adaptive and accurate tracking. It combines RFID tags, reference and tangible markers, and a camera. RFID tags were placed on physical objects and cards. The RFID reader reads the RFID tags for their mapping and linking to a virtual object. Virtual objects are superimposed on their visual markers. The camera tracks the manipulation with a physical object and generates true 3D virtual scenes by the service converter module. Augmented reality supports the reality of the virtual scene’s appearance with 3D graphics, allowing you to render a scene with a certain tangibility (see Figure 34). This solution is suitable for personal and casual users. They can easily interact with other users or virtual objects with this technology [58]. 

**Figure 35:**
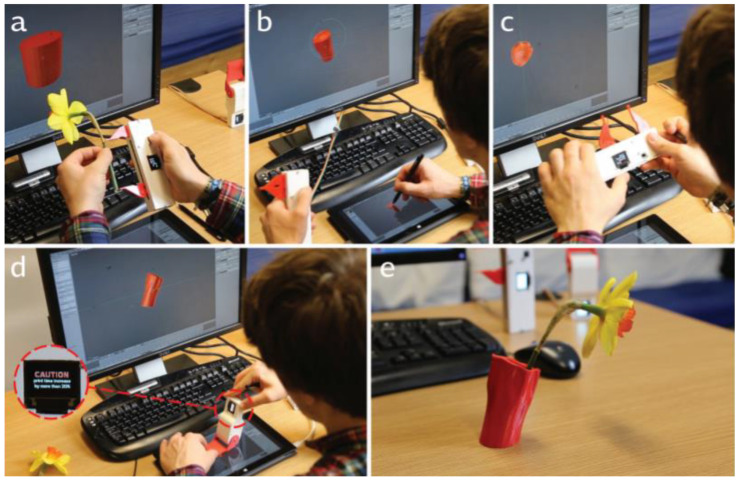
(**a**) creation of vase by using SPATA tools, (**b**) sculpting decorative features by SPATA tools, (**c**) checking the size of the model, (**d**) exploring flower hole angles, (**e**) printed object result [59].

Weichel et al. from Lancaster University developed SPATA. SPATA represents two spatial tangible measuring tools and a protractor and caliper. These tools can be used to design new objects. They can transfer a measurement into the digital design environment or it can help with the visualization of another measurement for a design decision. The SPATA Calliper measures the length, diameter, and depth of objects. It was realized by audio sliders as actuators. The voltage divider relative to the slider position was used for positional feedback. The SPATA Protractor measures the angle between two lines or surfaces. It was realized by a fixed beam and a blade, which can rotate around the center point. These components were attached to a Dynamixel AX-12A servo motor. The servo motor provides a serial interface and reports the orientation of the protractor. From the results, the process of design is more efficient and convenient than common tools. This approach is less error-prone [59]. Figure 35 shows the creation of a vase using SPATA calipers (a), sculpting decorative features by SPATA tools (b), checking the size of the model (c), exploring flower hole angles (d), printed object result (e).

### 3.7. TUI for Modeling in Architecture

Ishii et al. from MIT Media Laboratory proposed a tangible bit system consisting of metaDESK, transBOARD, and ambientROOM for the geospatial design of buildings. The technical concept of metaDESK consists of a tabletop, phicons (physical objects), passive LENS, active LENS, and instruments. ActiveLENS allow haptic interaction with 3D digital information bound to physical objects. Physical objects and instruments are sensed by an array of optical, mechanical, and electromagnetic field sensors. These sensors are embedded within the metaDESK. The ambientROOM is a supplement to Metadesk. In ambientROOM, ambient media are used to simulate light, shadow, sound, airflow, and water flow. The transBoard was implemented on a whiteboard called Softboard. Softboard is a product from Microfield Graphics, and it monitors the user’s activity of a tagged physical pen with a scanning infrared laser. Cards with bar codes were used in this implementation. RFID tag technology was used for scanning codes and identifying objects [60]. 

**Figure 36:**
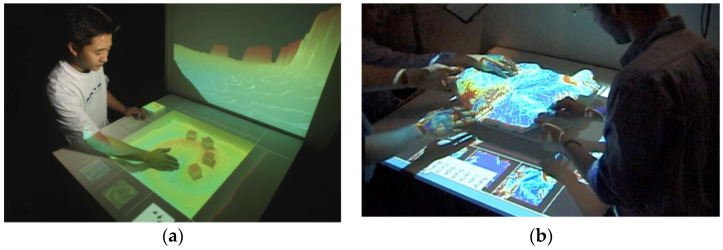
Manipulation with SandScape and projected onto the surface of sand in real-time (**a**) [61], Illuminating Clay in use (**b**) [62].

Piper et al. from MIT Media Laboratory proposed the first generation of a TUI for the design of architecture called Illuminating Clay. The technology represents physical models of architectural buildings. The models are used to configure and control basic urban shadow stimulation, light reflections, wind flows, and traffic jams. Using the TUI model, designers can identify and isolate shading problems and relocate buildings to avoid dark areas. They can maximize the amount of light between buildings. Building blocks and interactive tools are physical representations of digital information and computational functions. The problem with the first generation of TUI (Illuminating Clay) is that users must use a predefined finite set of building models. These models are defined physically and digitally (see Figure 36). Only the spatial relationship between them can be changed, not their form. Tangible Media Group proposed the second generation of the TUI [61,62]. 

Ishii et al. from MIT Media Laboratory proposed the second generation of TUI for the design of architecture called Sandscape (see Figure 36). A new type of organic tangible material (sand and clay) was used for the rapid modeling and design of a landscape. These flexible materials are integrated with fully flexible sensors and displays. This category of organic TUI has great potential to express an idea in material form. Landscape technology uses optical techniques to capture landscape geometry, while Illuminating Clay uses laser rangefinders to capture the geometry of a physical clay model [61,62,63]. 

Nasman et al. from Rensselaer Polytechnic Institute proposed a tool for the simulation of daylight in rooms. The technical concept consists of a digital pad, projector, and camera model of walls. Thus, creating a hybrid pad of the surface-computer interface. First, the architect inspects a real room after which he creates a physical model of the room. The room is visualized to evaluate the natural lighting at different times of the year. This system is better in terms of the architect’s deeper perception and understanding of the influence of light in terms of summer, winter, solstice, sunrise, sunset, and more. For the simulation of light propagation in space, a radiosity shadow volume hybrid rendering method is used. The system displays simulated lighting on the physical model using multiple projectors that are placed in the circle above the table [64,65]. 

Maquil et al. from the Luxembourg Institute of Science and Technology proposed a geospatial tangible user interface (GTUI) for Urban Planning. Current traditional tools for urban projects offer few opportunities for collaboration and design. This system consists of the following components: the PostGIS database (https://postgis.net/, accessed on 10 February 2021), a web map server, the reacTIVision computer vision framework, the TUIO Java client (https://www.tuio.org/?java, accessed on 10 February 2021), a GeoTools (https://geotools.org/, accessed on 10 February 2021) tabletop, wooden physical objects (circles, squares, rectangles, triangles), a camera, and music feedback for working with renewable energy. Maps are projected onto the tabletop from below. Physical objects have optical markers. These markers are detected by a camera mounted on the bottom of the table. These tools provide users with easy access to maps, intuitive work with objects, support offline interaction (positioning, tapping, etc.), and examine and analyze objects using physical manipulation. The concept of the GTUI provides new opportunities in urban logistics projects and determines the optimal placement of Urban Distribution Centres [66].

### 3.8. TUIs in Literature and Storytelling

Ha et al. from GIST U-VR Laboratory proposed the Digilog Book for the development of imagination and vocabulary. It combines the analog sensitivity of a paper book and digitizes the visual, audio, and haptic feedback of readers using augmented reality. The technical concept of the system consists of ARtalet, a USB camera, a display, speakers, and vibration. ARtalet computer vision processes input images from the camera that is mounted on the camera arm and captures 30 frames per second at a resolution of 640 × 480 pixels. ARtalet in Digilog Book presents the manipulation of the trajectory of 3D objects and deformation of the network in real-time, creating audio/vibration feedback to improve the user experience and interest. The osgART1 library was used to support the rendering of a structured graphical scene and tracking function based on computer vision. The user can create a scene from 3D objects from the menu and assign them audio haptic feedback. ARtalet allows the deformation of the physical grid, which the user can freely increase, decrease, and thus resize the 3D model. The user can rotate the object, manipulate it. The ARtalet can be used in the application for the creation of posters, pictures, newspapers, and boards. Another possibility is to use a TUI to create stories [67]. 

Smith et al. from CSIR Meraka Institute proposed a storytelling modality called StoryBeads and Input Surface. The BaNtwane people in South Africa used beads for storytelling and need a system for storing stories. The technical concept consists of physical objects (beads, self-made jewelry) with embedded RFID capsules called e-Beads. StoryTeller consists of a laptop, microphone, loudspeaker, and RFID reader. All these components are encapsulated in ‘the hides’ in the input surface looking like a rectangle table. The user puts an eBead without a story on the input surface, the RFID tag is scanned by the RFID reader, a prerecorded audio prompt invites the user to tell the story, the eBead is removed from the surface and the recording of the story is associated with the eBead. The story is played from loudspeakers when an eBead of the story is put on the input surface. This system was proposed for the BaNtwane people. The manipulation with beads and input surface were easy because they used components that they know. This system allows for preserving their cultural heritage. In the future, it can be used as a conceptual model of the Internet of Things [68].

**Figure 37:**
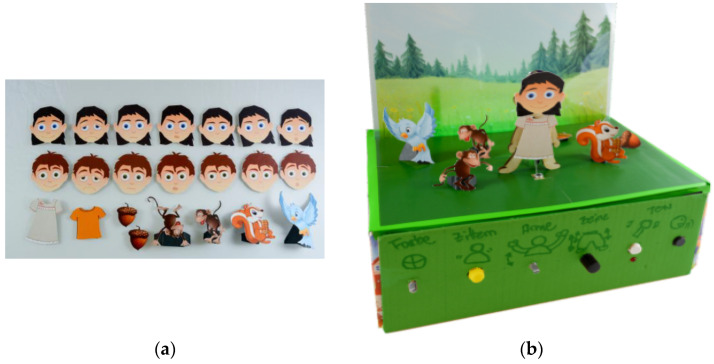
Interchangeable emotional faces, characters, and objects (**a**), interactive diarama for supporting storytelling (**b**) [69].

Wallbaum et al. from OFFIS—Institute for IT in Germany proposed a tangible kit for storytelling. The storytelling system was proposed to help children and their parents in exploring emotions. The technical concept of this system consists of a board with an embedded microphone and speakers, a servomotor and tactile motors, and the interaction controller, while there is a control panel outside of the board. On the board, there are interactive puppets and behind the board, there is an illuminated background. The child recreates scenes based on a storyline. To create the scene, the child uses the interactive puppets as male/female figures with different emotions, the characters of the house, background, scenery, scene elements, and figures of animals (see Figure 37). In the future, the storytelling kit can be more general, because each family has different routines and practices, so the emotions of a child are difficult to estimate. The storytelling kit is suitable for storytelling between parents and children [69]. 

**Figure 38:**
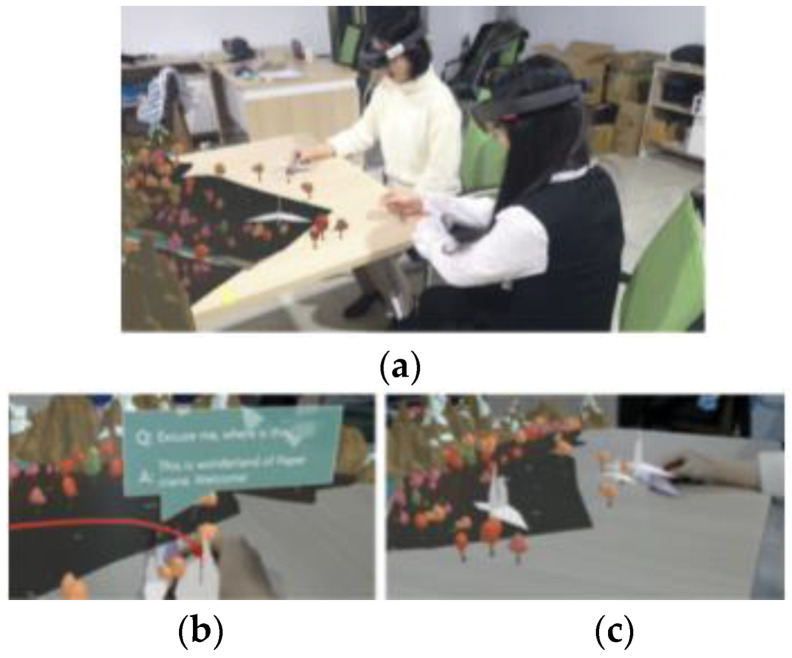
Storytelling model with two users view the story (**a**), story-teller’s views (**b**), the audience’s views (**c**) [70].

Song et al. from Shandong University proposed a TUI system for story creation by natural interaction. The technical concept consists of a desktop as an operating platform, while a PC allows hand data acquisition and gesture recognition by connecting the LM (Leap Motion) controller, with HoloLens glasses for coordinate mapping hand gesture positions and implementing them to the virtual scene to achieve tangible interaction. The Leap Motion is attached to the kickstand and detects the hand positions and gestures on the desktop. Through the HoloLens, the users see folding paper and learn to make origami. Then the user sees the virtual model of the origami. This is an easy way to create animation. The user can create the story, story viewing, recording the story, and storytelling to other users with HoloLens (see Figure 38). The function is switched by virtual buttons on the desktop and by the hand position recognition function. The system effectively supports children’s language skills and creativity and parent-child interaction [70].

### 3.9. Adjustable TUI Solution

The TUI approaches are described in this section because they were not tested in a concrete area, based on the classification of the areas (literature, modeling, education, etc.). The TUI approach for gesture recognition is described in this section. There is a general TUI implementation for manipulation with objects and pairing smart devices, too. This section presents a specific application in the aviation industry. The industry area was not classified in this review, so this approach is presented in this section because this is the only implementation.

**Figure 39:**
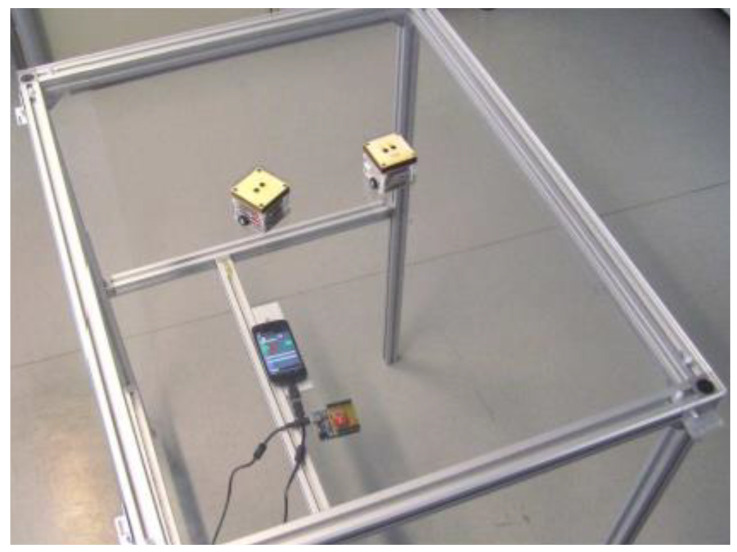
Two ACTOs, a smartphone, and an Arduino with a RF-module [71].

Vonach et al. from Vienna University of Technology proposed ACTO (Actuated Tangible User Interface Object). ACTO represents the modular design of a TUI with widgets. The ACTO hardware consists of these modules: an extension module, base module with RF-Unit, motor module, and marker panel. The main technical concept is divided into the surface with ACTO, Arduino with an RF-Unit, and the smartphone with WLAN. ACTO is compatible with the Arduino “Base Module” and RF communication capabilities that can control the ACTO positioning and rotating mechanism (see Figure 39). In the upper part, it is possible to connect a module for expansion, e.g., to graphic displays. The system communicates on smartphones with Android via WLAN. For example, these ACTO modules are used in an audio memory game, an expansion module with tilt sensors, rotation for controlling a music player, a module for measuring physical properties (light intensity, etc.), and an alternative motor module. From the initial evaluation, the system is suitable as a prototyping platform for TUI research. The system is also suitable for educational purposes to teach TUI concepts [71]. 

**Figure 40:**
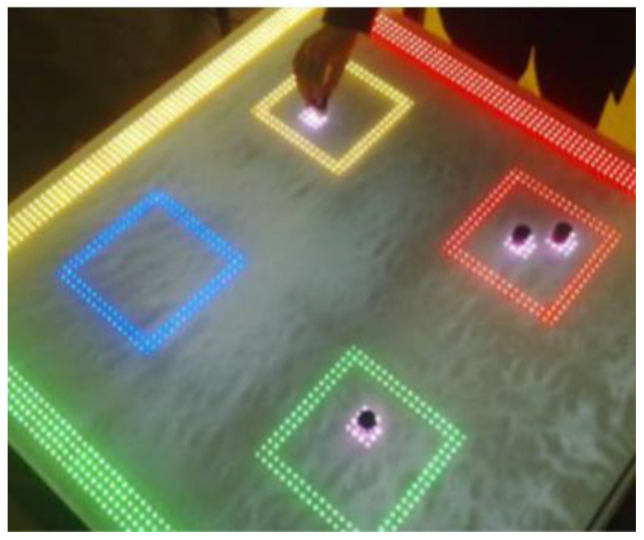
The Tangisense interactive table with using LEDs [72].

Kubicki et al. from University Lille Nord de France proposed the TangiSense table for interaction with tangible objects (see Figure 40). The TangiSense is a table that is equipped with RFID technology. By manipulating physical objects on the table, the user can simulate the operation according to the displacement of a material object. The technical concept consists of an RFID system (RFID tags), cameras for capturing physical objects, and a touch screen. Virtual objects are visual objects projected onto the table by LEDs that are placed on the table or video projector. Users can manipulate the virtual objects using the glove fitted with RFID tags because RFID tags are monitored by the camera. The camera is reliable, fast, and detects the position of the user’s fingers and objects in images. The implementation of the table using RFID is made of tiles with antennas on the surface. Each tile contains a DSP processor that reads the RFID antennas, the multiplexer antenna, and the communication process. The tiles are connected through the control interface to a PC via an Ethernet line. From these results, the interest in contactless technology and interactive tables is increasing [72]. 

Zappi et al. from DEIS University of Bologna developed Hidden Markov models for gesture recognition. Hidden Markov models can recognize gestures and actions using TUI to interact with the smart environment. Hidden Markov Models were implemented on a SMCube with a TUI. The technical concept of the SMCUbe consists of sensors (an accelerometer, 6 phototransistors), actuators (infrared LEDs), a microcontroller, and Bluetooth. The microcontroller samples and processes the data from the sensors and Bluetooth is for wireless communication with the PC. The results show the proposed technology can be implemented in smart objects for gesture recognition [73]. 

Henderson et al. from Columbia University proposed a combination of augmented reality and TUI opportunistic control in the aviation industry. A TUI was applied in an aircraft engine for simulating maintenance inspections using virtual buttons implemented as opportunistic controls and passive haptics. The technical concept consists of a laptop, camera, ARTag, widgets, and buttons with optical tags. The ARTag optical marker tracking library detects an array of camera frames. The camera is located above the user and tracks and recognizes the gestures of the user. The tasks were performed by the participants faster and were preferred to the basic method [74]. 

Lee et al. from Chonnam National University proposed a system for interaction with digital products. The technical concept consists of multi displays with a Wiimote camera and IR tangibles. IR tangibles are wands, cubes, rings with IR LEDs for interaction with displays. 3D coordinates of IR objects are calculated in real-time using stereo vision optical tracking. The Wiimote camera has been applied for infrared optical tracking IR tangibles and to capture user interactions and intentions. In this way, the user can easily manipulate and work with digital products using IR tangibles in a user-friendly environment with large displays and boards. The proposed system is available for use in a conference room, though low light conditions can be a problem [75]. 

Fong-Gong et al. from National Cheng Kung University Tainan proposed an interactive system for a smart kitchen. In the kitchen, there were moveable objects with different functions. Two cameras are situated above the kitchen table. The user can control the light, stereo, volume, and TV in the kitchen using tangible objects. Tangibles are plates, cups, and bowls with color patterns. The plate was used to trigger a corresponding list of music. A clockwise rotation on the plate means switching to the next song and a counterclockwise rotation means switching to the previous song. A cup was used to control the volume of the stereo and TV with a rotation of the cup clockwise and counterclockwise. The bowl was used for the configuration of ambient lighting. Bowls with different patterns control different lighting effects. The Speed Robust Feature (SURF) is an algorithm for pattern recognition on kitchen dishes. After removing the limitations, the smart home of the future can use systematic, identifiable patterns. Limitations in the research are lighting changes in the kitchen, the material of an object must be nonglossy to prevent interference detection [76].

**Figure 41:**
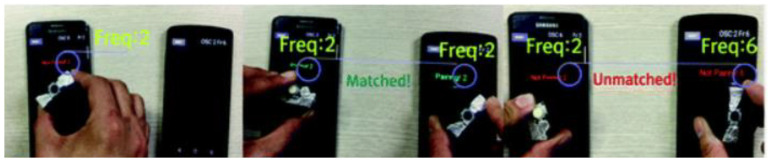
Application of tangible objects for pair smart devices [77].

Park et al. from Sungkyunkwan University proposed tangible objects for pairing between two smart devices. The user can pair smart devices with tangible objects which generate vibration frequencies and the application in the mobile detects the same frequency, and then the devices are paired. Vibration tangible tokens are composed of a small Arduino (Gemma), a coin shape linear vibration motor, a 3.7 V Li-ion battery with a capacity of 280 mAh, and buttons. Tangible objects are placed on the screen of the smart devices while they are running the application. When the frequencies are the same, the smart devices are paired and can broadcast, the data on other devices uses the Open Sound Control protocol (OSC) (see Figure 41). The advantage of this proposed system is the easy pairing of a device by a tangible process and users do not need to memorize the device name and ID. The process of authentication and target selection is reduced, too [77]. 

### 3.10. Commercial TUI Smart Toys

**Figure 42:**
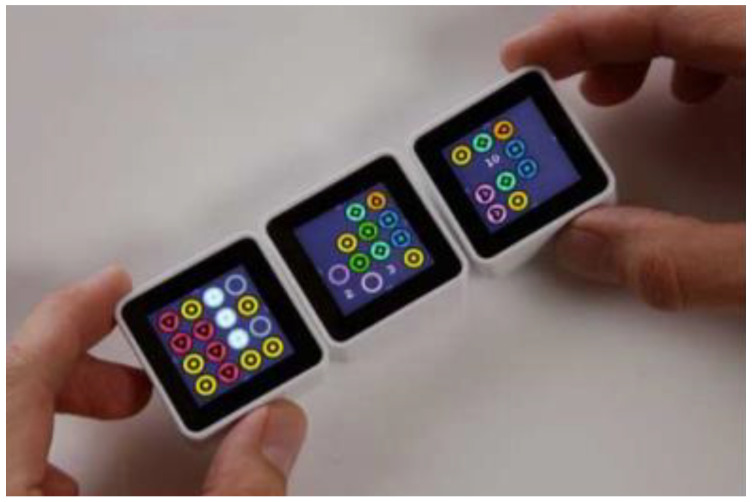
User plays arcade puzzler by Sifteo Cubes [48,78,79].

Merrill et al. from MIT Media Laboratory developed Siftables (or Sifteo Cubes). Siftables are commercially available tangible user interfaces. Siftables introduce a technology platform consisting of wireless sensor networks and a TUI. It allows a new approach to the human-computer system. It represents a physical manipulation for interacting with digital information and media. One way to use the TUI is in combination with a GUI, they include support for two-handed input. Another use of TUI is without a GUI, where physical objects directly embody digital information or media. The Sensor Network User Interface (SNUI) takes the form of a distributed TUI. In the SNUI, there are many small physical manipulators, enabling sensing, wireless communication, and user-controlled output functions. Motion sensors are placed on each side of the cube. These Sifteo cubes allow interaction with graphic displays. 1.5-inch blocks with clickable color LCDs are placed on each side of the cube (see Figure 42). These cubes can identify the location of the cubes. Each cube can work for 3 h, all 6 cubes are charged in the charging station, Users can play single and multiplayer games with Sifteo cubes [48,78,79]. 

## 4. TUI Technical Solution Analysis

The TUI technical solution can be divided into two basic approaches: sensors and image evaluation. Sensors capture data information. Image processing is based on a camera that captures the situation, whereupon the image algorithms process the information.

### 4.1. Sensory Technical Solution

The TUI approach uses a sensory technical solution consisting of a sensory system that captures the information from objects. Usually, microcontrollers process and evaluate this information and data.

The authors used a wide range of microprocessor platforms like ATMEL, TI, MicroChip Technology, and Diodes Incorporated. However, microcontrollers based on platforms like Arduino and Raspberry Pi are also used in TUI systems (see Table 2).

These technical solutions can be divided into two groups: communication and interaction between objects, communication between objects, and interaction with information on a board (table). Data can be processed in objects and give feedback or data are processed on a PC or laptop. 

#### 4.1.1. Wireless Technologies

Data can be transferred by commonly-used wireless technologies. The most often used wireless technology was the RFID (Radio Frequency Identification) technology. RFID technology consists of an RFID reader and RFID tags that are placed on TUI objects. The main function of RFID tags is the identification of objects. Each object (cards, cubes, phicons, etc.) has a unique ID. RFID tags represent tags for features (shape, color), position, and functions of objects. The objects used in the solutions reviewed were placed on a desk or tabletop and the RFID reader read the RFID tags and gave information to the microprocessor or laptop. Then the information was processed and evaluated or the loaded function was provided [16,29,30,33,42,43,58,64,68,72,80].

The second most used wireless technology was Bluetooth. Bluetooth was used only for data transmission, communication between objects and a PC or laptop [40,73,75]. Data were usually processed on a PC. The authors usually did not define the Bluetooth module type and standard versions. However, it was clear they used Bluetooth as a serial link wireless replacement.

The Wi-Fi standard was only used for data transmission in two recent works. Once as the standard for data transmission, a technology that enables wireless programming of an embedded system of the TUI [42,49].

The ZigBee standard was mentioned only once, it was used for measured data transmission [34]. Table 3 presents an overview of the wireless technologies used.

#### 4.1.2. Sensors

TUIs usually consist of sensors for the determination of motion accuracy, the position of objects, etc. The sensors that were used in the described solutions in the review are given below. 

Optical sensors such as infrared (IR) sensors were used for determining the relative position of the blocks [34], for measuring rotation [57], for detecting neighboring objects in close range [48,78,79], for tracking obstacles in the motion of the robot [42], and as actuators [73]. Infrared transceivers are tuned for extremely short-range communication (1 cm) [48,78,79].

Light-dependent resistors (LDR) or photoresistors were used to determine the relative position of the objects. The resistance of the resistor was changed depending on the intensity of the received light. These sensors provide information about the relative position of the two objects because if they are close enough, the LDR does not receive light [32,33]. 

Tilt sensors detect the motion of objects. They were used for the management of energy savings of TUIs. The TUI is in deep sleep mode until it is taken by the user [32,33]. Only the solution by [71] uses the tilt sensor as a tool for the determination of the number of the object’s tilts during usage.

Accelerometers, gyroscopes, magnetometers were used substantially for the detection of the motion of objects in the aforementioned TUI applications.

Most of these solutions were based on a three-axis accelerometer as a motion detector (lift, tilt, shake) of objects [32,33,34,49,57,73,78,79]. The accelerometer together with gyroscope and compass functionality and inertial sensors were used for more precise measurements of the movement pattern of the user when he or she places the objects [32].

The next approach for measuring the rotation angle of the object was based on using the Hall sensor. The application measured the rotation angle between the Hall sensor and a permanent magnet placed inside objects. A magnet was placed on the opposite side and less than a millimeter away from each sensor [57]. Table 4 presents an overview of the sensors used in technical solutions of TUIs.

#### 4.1.3. Feedback Possibilities

In the TUI approach, different feedback was used. Visual feedback was represented by a projector that projected color on tables. When the objects were placed correctly, the green color projects on the table of objects, while if the objects are placed incorrectly, the color turns red [5,13,14]. A combination of the projector and mirror allowed the projection of visual feedback onto the table when the projector was placed below the table [54]. The next visual feedback was green and red LED lights indicating when the user chose a symbol correctly or incorrectly [16].

Haptic feedback provided active feedback that was situated in a haptic glove. The glove provided vibration feedback through natural movement and positions [26]. The vibration actuator was situated in 3D objects, as well. The vibration pattern allows the user to easily understand. The vibration patterns consisted of various vibration waves such as a square wave, triangle wave, sin wave, and other shaped waves [67]. The next tactile feedback was a snapping mechanism linked to an anchor point, the object was physically shaped to the underlying grid [59]. Positional feedback was applied by a voltage divider relative to the slider’s position [59].

Audio feedback allows the playing of sound effects that the user can understand and easily manipulate objects [26,67]. Audio sliders used a dual H-bridge controlled through a custom PID controller which was implemented on the microcontroller [59]. 

The TUI systems sometimes combined multiple audio/visual/haptic feedback in one approach [67]. 

### 4.2. Image Processing

The technical concept of this approach was the scanning of tangible objects or the user’s work on tangible objects using a camera in real-time. The scanned image was processed by computer vision algorithms [5,13,16,41,67].

The main technical concept of image processing consists of a table or tabletop, a camera below or above the table, and tangible objects with markers.

In general, image processing systems can be divided into markerless and marker approaches. The camera can recognize the markers or pictures on objects and the position of the objects. The camera can be placed below the table for capturing the fiducial markers or attached above the table for detecting the markers. It depends on the construction of the TUI technical solution.

#### 4.2.1. Marker Approach for Object Recognition

Tangible objects have markers which are unique for each object. Marker systems used visible markers or hidden markers. 

Markers defined in a TUI system colors, shapes, functions, animals, musical instruments, buildings, etc., and indicated different heights of objects, etc. In the works, there were markers specified as fiducial markers. Fiducial markers can be printed on paper or different materials and placed on tangible objects. It can be in color or black/white. Some works used fiducial markers from the library of computer vision approaches (ARtag, ARToolkit). They were black/white markers in a square [5,10,14,16,23,28,45,51,54,55,64]. 

In some approaches, they used fiducial tracking of infrared signals from IR LEDs or by shining IR light generated by an LED array on a reflective marker attached to the user’s hand [9,26,54,55]. 

#### 4.2.2. Markerless Approach for Object Recognition

The markerless system recognizes pictures with different colors, shapes. The pictures were printed in color or black/white. The images look like arrows or different shapes, pictures of animals, or with different objects or different colors [34,64,81]. 

#### 4.2.3. Used Cameras for Image Processing

However, in each technical solution of image processing, there is a situation table, a tabletop where users manipulate tangible objects. The camera was situated above the table or below the table, it depended on the construction of the TUI system. In the first approach, cameras were used for capturing the situation, and images were sent to a PC for image processing by computer vision platforms. In the second approach, cameras are used to capture the situation and process the image in the camera because they include the image processing algorithm, such as the Wiimote camera [9,75]. 

For image capturing situations, webcams [16,23,28,29,31,45] were used in the work of [28], specifically, the Logitech Webcam Pro 9000, USB cameras with a resolution of 640 × 480 were used in [67], in [74], specifically, the Point Grey Firefly MV was used, infrared cameras in [9,26,54], and in the work by [75], specifically the Wiimote infrared camera with a resolution of 1024 × 628. In the work of [55], a Sony PS3 Eye camera was used without an infrared filter and an infrared light pass filter 850nm was added. In the following works, there was no type of camera specified [13,18,41,58,64,66]. In the work of [13], a camera with a resolution of 1280 × 960 was used. 

#### 4.2.4. Used Computer Vision Platforms for TUI Applications

The cameras used algorithms for automatic object recognition. Object recognition is the technique of computer vision for identifying objects in images and video [5,14]. The open-source systems for computer vision were OpenCV, ARToolkit, ARTag, reacTIVision, osgART, library1.

ReacTIVision is an open-source platform for computer vision. It is used for the fast and robust tracking of fiducial markers placed on tangible objects and their identification in real-time [9,23,25,26,45,51,55,66]. The principle of the software is the binarization of the input images with an adaptive thresholding algorithm. This image is then segmented into a region adjacency graph (tree sequence). Unique left heavy depth sequences were found in this graph. These sequences were encoded into fiducial symbols. In the last step, the found sequences were matched against a dictionary to get a unique ID number. This concept allows the efficient calculation of the fiducial marker’s orientation and center point determination of identity, and to transmit this to the client applications. When the user’s movement is fast or markers are not recognized, reacTIVision used a shape-matching algorithm. This algorithm uses the result of image segmentation to retrieve and identify fingertips on the surface from possible region candidates [12].

ARTag is an augmented reality system for imaging 3D virtual objects, games, animations in real-time with video. It was inspired by ARToolkit. ARTag allows the use of square ARTag markers, which are placed on objects. The camera was tracking the ARTag optical marker tracking library by the detection of a fiducial array in the camera’s frame. The computer vision algorithm can calculate the camera’s coordinate system in real-time. Computer graphics give the illusion that 3D animations or video games appear in the real world. The segmentation algorithm ignores the depth information in the camera’s frame. First, the physical model was defined as a convex polyhedron using 3D points, which were placed in a common physical interface coordinate system. The algorithm allows the conversion of coordinates in the gesture space (camera coordinates) to and from physical interface coordinates [74].

ARToolkit is an open-source computer tracking library. The algorithm can calculate the real camera position and orientation of square physical markers in real-time. It solves viewpoint tracking and virtual object interaction [28]. The principle of this software is the camera first captures video images. Then the images are binarized and the markers are searched. The position and orientation of the fiducial markers are calculated relatively to the camera. Then a matching algorithm identifies the markers, as a matched symbol inside the marker with templates in memory. The marker IDs are known and the 3D virtual objects are aligned with the marker and rendered in a video frame [27].

OpenCV is an Open Source Computer Vision and machine learning software library. The library contains more than 2500 optimized algorithms to detect and recognize faces, identify objects, classify human actions [75], videos, track camera movements, track moving objects, extract 3D models of objects, find similar images from an image database, etc. [17]. This software was used for real-time image processing of the captured image of tangible objects (pens) with markers. OpenCV calculated and drew lines from the central location and calculated the angle of the lines. Then the output is graphically displayed by a projector on a table [16].

The next type of image recognition used IR signals. The infrared signal is detected by the Wiimote camera. The wrj4P5 interprets the infrared signal which was detected by the Wiimote [9]. Wrj4P5 is a library for programming environment processing. It is the model of the Wii Remote and its extensions. The stereo vision technique is used for the calculation of 3D coordinates of the IR tangible objects. The Wiimote camera tracks the IR tangible and generates graphics corresponding to the user’s movement [75]. The next algorithm for image processing from the infrared image was used in [54]. The image processing of infrared images was based on the K-means method of the nearest neighbor (KNN) using nine features.

The context-adaptive tracking method was used for adaptive vision tracking. This method adjusts the locations of invisible markers by interpolating the locations of existing reference markers and previous ones and removes the jumping effect of movable virtual objects. The vision tracking technique processes the camera’s frames and recognizes the locations of markers [58].

Color markers on the tangible objects (walls) indicate their height, doors, and windows. The scene is captured by a camera and based on computer vision techniques, the color-coded objects are recognized and the algorithm can automatically interpret the physical elements to 3D digital models [64]. 

Fast Lattice shape matching (FastLSM) is an algorithm for the real-time computation of the deformation of physical objects to virtual 3D objects. The algorithm divides the region into many sub-summation regions and calculates the movements of particles separately. The movements of the particle (object) are calculated followed by the translations and rotations of the shape-matching regions. The algorithm is available for relatively complex models. Free form deformation (FFD) is a method for connecting the deformation of the lattice model with the deformation of the virtual model. The osgART library1 was used for rendering the graphical model’s scene used for computer vision tracking functions. Computer vision was applied to the captured images where the user works with tangible objects. The tangible objects include fiducial markers on the top [67]. 

## 5. Discussion

The tangible user interface is used in diverse areas such as architecture, methods for learning, medicine, and psychology, programming robots, construction of database queries, compilations of music, modeling of 3D objects, gesture recognition, in smart toys, modeling in architecture, and in literature and storytelling (see Section 3). 

Each of the identified application areas could be represented by typical examples. Unfortunately, most of them are in development now, and they are not widely used. Even though it was shown that a tangible user interface is better for users than a graphical user interface based on knowledge—the TUI is more interesting for users, more realistic, offers feedback, and a better understanding of abstract concepts in the learning process. It turns out that it undoubtedly has its place in the field of education, whether it is a standard subject at different levels of education. The most published articles are in the field of education of traditional subjects but also in programming, which is very popular now. TUIs achieved better results with children and they are more interested in programming in this form. We must therefore ask ourselves why TUIs are not widely used in education when they have such good educational results. There can be several reasons, from the rigidity of education systems, insufficient information of teachers, the age of the teaching staff, or is it a question of financial demand? These questions can only be answered by researchers in the field of pedagogy and will certainly vary from country to country. 

A very interesting application area of TUI is medicine and psychology. This is because patients and people with various disabilities do not have to be able to work with a personal computer, which is the most widely used technology. Then TUIs become both a means of rehabilitation and a means of diagnosing the disease and diagnosing the success of rehabilitation therapy. As biomedical engineers, the field of medicine is very close to us and we also see hidden problems in TUI applications in this industry. The biggest advantages are the great variability of the solutions, ease of use for professional staff, and patient safety, which is defined by many strict standards. One of the most limiting in the development of TUI seems to be technology. The computing power is not a limiting indicator in the use of TUIs nowadays. What is limiting is the way to acquire information about the movement and position of the TUI in the real world and its transfer into virtual space. We can split the typical technical approaches into two different methods. The first way is a sensory technical solution and the second way is scanning by a camera and further image processing based on reference marks. The sensory technical solution uses many different sensors for gathering information and further processing widely used in industry. There are simple sensors used, like light-dependent resistors or photoresistors, followed by more complex sensors like tilt sensors, accelerometers, gyroscopes, and magnetometers. These are used for the detection of motion of objects in the proposed approaches in the review and they require smarter signal processing, usually hidden in the microprocessor platforms. The used signal processing method is not usually presented. The authors do not consider it important to introduce it in more detail, although the choice of the data processing method will fundamentally affect the quality of the TUI functionality. The authors usually do not focus on the technical limitations of the chosen sensors—electrical mechanical drift influence from the environment. Most of the reviewed papers do not mention the settings of the environment, the signal preprocessing made in sensors. This is a hidden problem, which prevents the greater use of sensory TUIs in real applications.

One of the promising technical solutions is wireless technologies for the identification of TUIs. The most commonly-used technology was RFID technology, which gives very nice results for object recognition. It is followed by low-power short-range technologies like ZigBee and Bluetooth. The use of Wi-Fi is rare, which makes sense, given the energy requirements of this technology. Infrared communication is rarely used, it has advantages in comparison to other wireless technologies because it enables data transmission between TUI objects and provides wireless identification. 

A separate issue that must be discussed in the field of TUI technical solutions is the method of their power supply and their energy independence. The choice of battery is complicated because the objects are smaller, they can be grasped by hand. Therefore, it is necessary to use a battery with smaller dimensions. It is the size of the object that affects the choice of battery and thus limits the choice of the size of the battery capacity. For choice, the battery is necessarily approached so that other priorities do not suffer from the capacity of battery life such as battery size, radio transmission, communication frequency, size of components, and price. The use of ultrasmall components can cause TUI objects to need a more specialized and therefore more expensive assembly. Therefore, it is better to avoid them and choose larger components, but this will also result in larger objects [33,34,56,71,78].

The size, performance, and price of the used components are resolved for sensor solutions. This is a more challenging constructional process for a designer hardware solution. The obtained data are sent to the superior system. Image processing works with open-source platforms for computer vision for objects with markers or images which are processed by software. Based on the data obtained from both types of the TUI approach, it is possible to obtain quantitative and qualitative information/data, that is, to evaluate the user and his work with objects efficiently, objectively, and with time savings. The data are evaluated by software or manually by the teacher/doctor/psychologist/therapist [33,34,57].

The second way is image processing. In this way, the camera captures the image and then the image is processed by a computer vision algorithm. For capturing images or videos, they use an infrared camera, web camera, USB camera, and Wiimote camera. Sometimes camera-based commercial solutions for position detection like Kinect are used. However, the camera can also be used just for recording the situation, for monitoring the progress of therapy and their evaluation by teachers and therapists. The camera can be attached underneath or above the table where the user manipulates objects, markers on the object can be required or not, depending on the technical solution. Markers are a part of an object and can be hidden, visible, or invisible. Visible markers can be defined from a library of computer vision. Invisible markers represent IR signals from IR LEDs. The marker system usually recognizes images with different colors and shapes. Thanks to computer vision and image processing, situations are processed on a PC/laptop using open-source algorithms. ReacTIVision, OpenCV, ARToolkit, ARTag, and osgART library1 are used as an open-source software for computer vision with the aim of image recognition. The use of cameras transmits the complexity of TUI identification on a personal computer. Algorithms for image recognition are significantly more computationally intensive than algorithms for sensory solutions. Unfortunately, the authors of articles on TUI do not pay enough attention to the selection of specific algorithms for image processing and, as with sensory solutions, they are black boxes. The reviewed paper also shows the main limitation of the camera-based solution—“the shielding”. Once one object is shielded for a second, it is not possible to precisely detect its position. To solve this limitation, we must use multiple cameras or define that the TUI will only be used in 2D space or will not stack on top of each other. We also need to define the acquiring scene in time slots without hands. 

A problem with image processing can be tracking markers complicated by a large distance to the table or a poor lighting situation. An innovative approach could be laser-based marker tracking, which would not be affected by the quality of the lighting in the environment. For better object detection, it is possible to apply Arduino to each object in the future and connect to a superior system via Bluetooth, where the camera monitors the area [28,71].

The next large advantage of TUIs in comparison to GUIs is the possibility of feedback. Of course, there are existing solutions for optical and audio feedback. However, the haptic option is only possible for TUI. The problem of incorporating haptic feedback is increasing the object weight due to the use of a motor and filling the space inside the object with other components. Of course, adding feedback will also increase the cost of implementing the object. We also identified the use of a Peltier heat pump as a very interesting method of feedback. This is an electronic component that can heat or cool the object using an electric current. Objects would be cooled, which would allow for feedback [28,57,73,78]. 

Based on the review, it is clear that the most common application area of a TUI is in the field of education, although basically each of the described areas of application aims to engage the user and, in some way, educate or develop.

A TUI tries to bring the taught problematics as close as possible to the user, by making the given problem graspable, in a way that can be visualized and better presented. This is a significant trend in education today because children, students, and adults in education through play have a higher motivation for further work. TUIs thus follow trends, abandoning classical learning, in which children are placed with memorized learning demands.

An important and very promising area of TUI development is the field of medicine, mainly in fields focused on the nervous system, brain, psychology, and psychiatry. By materializing the virtual world while providing many more sensory perceptions, TUIs are the most appropriate means of developing brain neuroplasticity and generally supporting the proper functioning of the nervous system. In addition, they make it possible to monitor and measure the activities performed. Thanks to that it is possible for a very early evaluation of the whole treatment process. We can say that TUI combines the features of a smart rehabilitation tool and secretly also a diagnostic tool. Thus, the rehabilitation process can then be performed efficiently and correctly not only at rehabilitation clinics under the supervision of a specialist but also at home. Professional medical staff can more often and better evaluate the course of the rehabilitation process, or adapt it to the current requirements of the patient.

The latest trend is the connection of TUI with augmented reality. When TUI objects become a functional part of virtual space while preserving natural shapes that correspond to those in virtual reality. This opens up another area of application in the gaming and entertainment industry.

## 6. Conclusions

This review article presents an extensive overview of the applicability of a tangible user interface in many areas with a focus on its technical solutions. The TUI applies to a wide area. For this reason, the articles were divided into sections according to the application area. We have identified eight basic areas, including the teaching of traditional subjects, applications in programming, and database development. These areas have large commercial potential because they have a high motivational potential for students. An important application area is in medicine and psychology, which seems to be very promising in the future. TUIs have their applications in assistive technologies, which are growing now. An interesting area is applications in art, where TUIs can be used to compose music or construct novels. TUIs are usable in engineering, like designing complex architectural solutions. In general, TUIs have a great advantage in applications where they are formed in 3D space, like 3D mechanical movable models, 3D scenes for augmented reality or aviation, sensorics, and models. 

The second part of the review presents the technical concepts of TUIs. The technical solution can be divided into two different methods. The first method is a sensory technical solution and the second is scanning by a camera and further image processing based on reference marks, for example. 

The sensory technical solution consists of many sensors for gathering information and further processing. Wireless technology can be used for transmitting information to the main component. The main component (a PC, laptop, a microchip in an object or tablet) processes data from the objects and gives feedback to the user. Feedback can be visual, haptic, audio, alone, or in combination. 

The second way is image processing, where an infrared camera, web camera, or USB camera was situated above the workspace or underneath the table. Objects have reference marks for the identification of objects. Thanks to this, computer vision and image processing evaluates the situation. Open-source systems were used for computer vision.

The review identifies the limitations of today’s TUI solutions from the technological point of view and shows them to be barriers against the greater application in practice.

## Figures and Tables

**Table 1:** The most common TUI application areas.

TUI Application Area	Short Description
Teaching	Stimulation of the learning process in traditional study subjects Increase of attractivity in different education levels (school, kindergarten, and exhibitions).
Medicine and psychology	Solutions for blind people, children with ADHD (Attention Deficit Hyperactivity Disorder), or with Down Syndrome Early detection of motoric impairments in childhood and detecting developmental delays in children.
Programming and controlling robots	Understanding the basics of programming or controlling robots for children, students, and visually impaired persons.
Database development	Development of database queries by tangible blocks
Music and Arts	Compilation of music
Modeling of 3D objects	Augmented reality and TUI design of 3D objects by tangible measuring tools.
Modeling in architecture	Geospatial design of buildings Configuration and control of basic urban shadow simulation, light reflection, wind flows, traffic jams, for simulation of daylight in rooms.
Literature and storytelling	Development of imagination and vocabulary and storing stories of cultural heritage

**Table 2:** Used microprocessor platforms.

Technology	Function	References
Atmega328p	microcontroller	[33]
ATMega 168	microcontroller	[73]
Rasberry Pi	microcontroller	[33]
Arduino	microcontroller	[8,11,16,41,42,44,71,77]

**Table 3:** Used wireless technologies.

Technology	Used with	References
RFID	RFID reader, RFID tag	[16,29,30,33,42,43,58,64,68,72,80]
ZigBee Module		[34]
Blue Giga WT12	Bluetooth 2.0 transmitter	[40,73]
NRF24L01+mini	RF device	[39]

**Table 4:** Used sensors in technical solutions of TUIs.

Sensor	Used as	References
Optical sensor	Determining the relative position	[34,42,48,57,73,78,79]
Accelerometers, gyroscopes, magnetometers	Motion measurement	[32,33,34,49,57,73,78,79]
RFID sensors	Antenna, RFID chip	[16,29,30,33,42,43,58,64,68,72,80]
Hall sensor		[57]
Light-dependent resistor	Determining the relative position	[32,33]
Tilt sensor	detect the motion of objects for the management of energy savings of TUIs	[32,33,71]
